# Supporting in situ conservation of the genetic diversity of crop wild relatives using genomic technologies

**DOI:** 10.1111/mec.16402

**Published:** 2022-02-25

**Authors:** Peterson W. Wambugu, Robert Henry

**Affiliations:** ^1^ Kenya Agricultural and Livestock Research Organization Genetic Resources Research Institute Nairobi Kenya; ^2^ Queensland Alliance for Agriculture and Food Innovation University of Queensland Brisbane Queensland Australia; ^3^ ARC Centre of Excellence for Plant Success in Nature and Agriculture University of Queensland Brisbane Queensland Australia

**Keywords:** crop wild relatives, genetic diversity, genomics, in situ conservation, plant genetic resources

## Abstract

The last decade has witnessed huge technological advances in genomics, particularly in DNA sequencing. Here, we review the actual and potential application of genomics in supporting in situ conservation of crop wild relatives (CWRs). In addition to helping in prioritization of protection of CWR taxa and in situ conservation sites, genome analysis is allowing the identification of novel alleles that need to be prioritized for conservation. Genomics is enabling the identification of potential sources of important adaptive traits that can guide the establishment or enrichment of in situ genetic reserves. Genomic tools also have the potential for developing a robust framework for monitoring and reporting genome‐based indicators of genetic diversity changes associated with factors such as land use or climate change. These tools have been demonstrated to have an important role in managing the conservation of populations, supporting sustainable access and utilization of CWR diversity, enhancing accelerated domestication of new crops and forensic genomics thus preventing misappropriation of genetic resources. Despite this great potential, many policy makers and conservation managers have failed to recognize and appreciate the need to accelerate the application of genomics to support the conservation and management of biodiversity in CWRs to underpin global food security. Funding and inadequate genomic expertise among conservation practitioners also remain major hindrances to the widespread application of genomics in conservation.

## 1 INTRODUCTION

Crop wild relatives (CWRs) play a critical role in ensuring food and nutritional security, economic development as well as environmental sustainability. It is estimated that there are about 50,000–60,000 CWR species globally, out of which 700 need to be prioritized for conservation (Maxted & Kell, 2009). Some of these CWRs and wild food plants are usually harvested and used as food and source of income, for example during periods of drought and food scarcity. More importantly, they are a source of readily available genetic variation for crop improvement. CWRs typically contain more diversity than domesticated crops as they still retain most of the genetic variation lost through various domestication related bottlenecks. Owing to their adaptation to diverse range of habitats, they are potential source of novel genes and alleles for adapting crops to the ever growing problem of climate change (Hajjar & Hodgkin, 2007). Although there has been increased interest in the conservation and use of CWRs, they still remain underutilized in crop improvement mainly due to inadequate conservation efforts (Vincent et al., 2013). CWRs are increasingly facing natural and anthropogenic‐related threats in their natural habitats leading to continuous genetic erosion and even extinctions (Bilz et al., 2011; Jarvis et al., 2008). In situ conservation has over the years emerged and been recognized globally as an important approach that complements ex situ conservation in safeguarding the world’s plant genetic resources. However, compared to ex situ conservation, in situ conservation is relatively poorly developed and receives much less attention.

**FIGURE 1:**
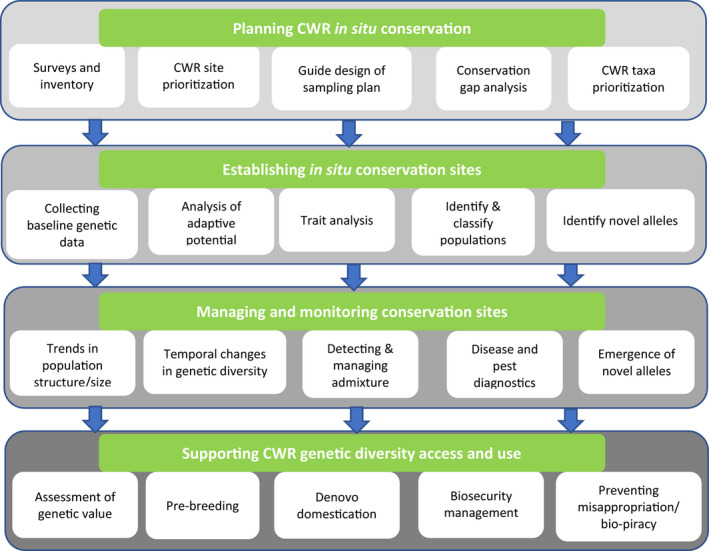
Application of genomics in genetic resources conservation

Conservation of plant genetic resources has over the years greatly benefited from the use of molecular based approaches. The last decade has seen major advances in genomics particularly in DNA sequencing which have significantly increased resolution in conservation genetic studies. There has however been debate on the rationale of using genomic approaches while the traditional genetic methods can address most of the conservation related issues (McMahon et al., 2014). Previously, conservation genetics has mostly made use of only a few markers to make inferences and inform decision making on conservation practice. As will be highlighted in this study, there are many instances where the earlier molecular technologies have failed to adequately resolve critical conservation issues thereby leading to conflicts and contradictions. The line that distinguishes genetics from genomics is in some cases somewhat blurred and their distinction varies between studies (Flanagan et al., 2018; Supple & Shapiro, 2018). In this review, we consider genomic studies as those analysing whole genome data or those that use thousands of markers to probe the entire genome. We review the actual and potential application of genomics in supporting in situ CWR conservation (Figure 1). Major progress has been made in the development of diverse genomic resources for some CWRs. Reference genome sequences are now available for a growing number CWRs (Brozynska et al., 2015) but many more remain uncharacterized. Genomics has created enormous opportunities that are enabling greater, efficient and more targeted utilization and conservation of these genetic resources. Recent large scale projects aim to coordinate the capture of genomic information for all life forms (Exposito‐Alonso et al., 2020), with an emphasis on capturing genomic data before biodiversity is lost. Rare and endangered species have been a focus of such efforts. CWRs are another group that deserve special attention.

## 2 OVERVIEW OF THE STATUS OF CONSERVATION OF CROP WILD RELATIVES

Despite the importance of CWRs, a review of literature paints a picture of neglect in the conservation of these valuable genetic resources. Substantial efforts and progress have been made in the ex situ conservation of plant genetic resources, yet CWRs remain grossly under‐represented in genebanks around the world. About a decade ago, it was estimated that out of the more than 7.4 accessions conserved in global genebanks (FAO, 2010), only about 2%–6% constituted CWRs (Maxted & Kell, 2009). The number of CWRs having accessions in seed banks was estimated at 6% (Maxted & Kell, 2009). However, with the recent global conservation efforts spearheaded by various institutions such as the Global Crop Diversity Trust (Dempewolf et al., 2014), aimed at improving the conservation and use of CWRs, their conservation status may have changed positively. The current in situ and ex situ CWR conservation status is likely to be known with the publication of the third state of the world report on Plant Genetic Resources for Food and Agriculture (PGRFA) whose preparation is underway.

Numerous studies have reported that even where CWRs exist in genebanks, their diversity is poorly represented, thus calling for systematic collecting efforts (Castañeda‐Álvarez et al., 2016; Khoury et al., 2019; Syfert et al., 2016). These species are better represented in in situ protected areas than in seed banks. However, even in cases where CWR populations occur in protected areas, they usually do not receive any active management and their existence and survival is just a product of the establishment of those areas (Wambugu et al., 2013). Where in situ conservation programmes exist, they are severely limited in geographical coverage. This situation seems to be somehow changing as countries are increasingly developing targeted national in situ conservation plans for CWRs (Fielder et al., 2015; Labokas et al., 2018; Phillips, 2017; Taylor et al., 2017; Teso et al., 2018). Globally, botanic gardens play an important role in CWR conservation.

Compared to ex situ conservation where a lot more information is available about the costs involved (Koo et al., 2003; Pardey et al., 2001), little is known on the costs of establishing and maintaining in situ conservation genetic reserves. There is also paucity of information on the costs of using genomics in supporting in situ conservation. However, even with the decreasing costs of genomic technologies, the costs of using genomics, for example in demographic population monitoring, still remain higher than those of traditional genetic approaches.

## 3 CONSERVATION GAP ANALYSIS

A robust conservation gap analysis is important in guiding the development and implementation of an effective conservation plan. Depending on objectives and priorities of the conservation programme, this analysis can be done at different levels namely genetic, taxon, ecogeographical and trait level using different approaches. While conservation initiatives aim and ensure conservation of as much taxonomic, genetic and ecosystem diversity as possible, gaps have been noted. The analysis of gene level diversity and its conservation status as well as its actual conservation has been neglected (Laikre, 2010). In in situ conservation, genetic diversity has largely been overlooked as focus has mainly been on populations, species, habitats and ecosystems (Coates et al., 2018). Phylogenetic and functional diversity is rarely considered during establishment of conservation sites (Cadotte & Tucker, 2018). As will be discussed later in this paper, genomics has potential for undertaking robust monitoring of levels and patterns of genetic diversity as well as assessing conservation status at genome scale. At the trait level, genomic analysis is important in determining whether the genotypes of cultivated and wild genetic species possess target traits. This knowledge is useful in assessing whether important functional traits are adequately conserved in existing populations or germplasm collections. Genomic tools can be used to complement geographic information system (GIS) and ecology‐based predictive characterization approaches such as focused identification of germplasm strategy (Bari et al., 2012; Bhullar et al., 2009; Endresen et al., 2012) to identify populations likely to possess important adaptive traits. Identification of such traits is important in supporting trait based conservation.

Taxon and geographical level in situ conservation gap analysis can be conducted by comparing in situ diversity with that represented in herbarium samples, ex situ collection or total diversity inherent in a taxa (Maxted et al., 2008). However, the natural diversity inherent within a taxa is usually not known. Whole genome‐ based population genomic studies which are continually being conducted for many CWRs (Baute, 2015; Wang et al., 2014) are increasingly providing information on the molecular genetic diversity inherent in a population or taxa. In order to comprehensively capture the available genetic variation, wide sampling from the species range is recommended, including analysis of ex situ collections. Large scale sequencing efforts such as the 3,000 rice and chick pea genome projects (Varshney et al., 2021; Wang et al., 2018) are contributing to better understanding of genetic variation present in a taxa. With increased availability of information on genetic variation inherent in a taxa, genomics can potentially provide a tool to assess the threat status of a taxa or population based on levels and patterns of allelic diversity. Such assessment is likely to be relatively easy to conduct in case of localized populations or taxa as compared to those that are expansively distributed.

## 4 PRIORITIZATION OF CWR TAXA FOR CONSERVATION

Due to the high number of CWRs that need protection, the budgetary requirements exceed the resources available for most conservation initiatives. Conservation managers therefore need to undertake an objective prioritization of taxa to be conserved (Maxted et al., 2006; Meilleur & Hodgkin, 2004). Prioritization is based on several criteria, key among them being relatedness between the CWR and the domesticated crop, economic value, value to breeders, endemicity, level of threat that the taxa is facing and their importance to national as well as global food security (Maxted & Kell, 2009; Phillips, 2017; Teso et al., 2018). In addition to this taxonomic based prioritization, conservation practitioners may set priorities based on functional diversity.

**FIGURE 2:**
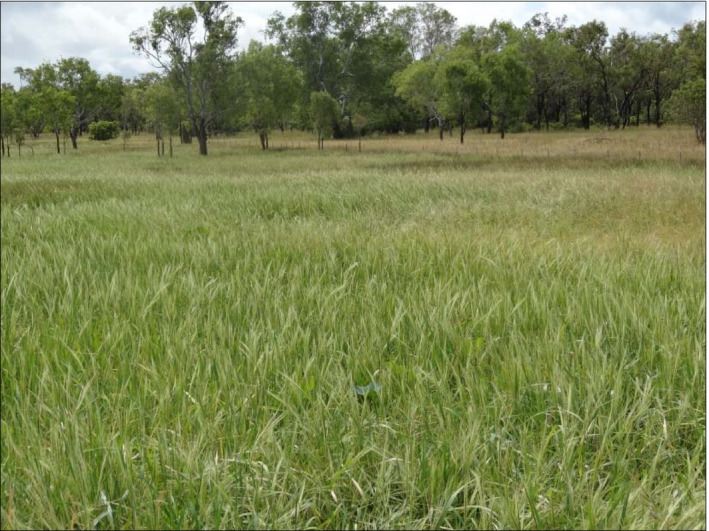
In situ populations of Australian wild rice in Lakefield National Park, North Queensland, Australia. Genome analysis has identified novel genetic diversity among these populations

Several concepts which define the genetic and taxonomic relationships between the cultivated and wild species have been developed and form the principal prioritization criteria. These include the gene pool concept (Harlan & de Wet, 1971), taxon group (TG) and provisional gene pool (PGP) concepts (Maxted et al., 2006; Vincent et al., 2013). Based on the gene pool concept (Harlan & de Wet, 1971), the primary gene pool which is the closest to the crop and whose genes are most accessible for crop improvement should be given priority, followed by secondary and finally the tertiary gene pool in that order. Analysis of genome data in the *Lens* genus identified four gene pools which are consistent with compatibility patterns among lentil (*Lens culinaris* Medik) and its CWRS, thus resolving the gene pool classification in this genus which has remained inconsistent and contradictory (Wong et al., 2015). Genome sequencing is enabling assessment of the novelty of genetic resources thereby enabling their prioritization in conservation (Henry, 2013). Genomics has enabled identification of novel wild rice genetic resources in Australia and confirmed their distinctness from Asian wild rice (Waters et al., 2012) (Figure 2). Although molecular markers have greatly helped in defining genetic relationships between CWR and domesticated crops, these have in some cases remained inconclusive, contradictory or ambiguous (Baute et al., 2016; Hwang et al., 2019). Most phylogenetic studies aimed at providing insights on genetic relationships have resulted in varying levels of phylogenetic discordance. Whole genome data is providing an opportunity to resolve previously intractable phylogenetic relationships (Stein et al., 2018). Genome wide Genotyping by Sequencing data has provided a refined phylogeny of the *Helianthus* genus, revealing a close genetic relationship between cultivated sunflower and *Helianthus winteri* J. C. Stebbins, a newly described sunflower wild relative (Baute et al., 2016). Chloroplast genome analysis has also been used in reconstructing phylogenetic relationships between wild and domesticated taxa in the *Oryza* AA genome group (Brozynska et al., 2014; Wambugu et al., 2015) and other genera including *Citrus* (Carbonell‐Caballero et al., 2015), *Sorghum* (Song et al., 2019), *Allium* (Huo et al., 2019) and *Musa* (Zhang et al., 2019). Compared to nuclear genome data, chloroplast genomes possess traits that offer particular advantages in studying phylogenetic relationships (Takahashi et al., 2008) (Small et al., 2004). The analysis of more loci in phylogenomic analysis results in increased resolution compared to traditional phylogenetic methods.

The taxon group concept is an alternative taxonomic‐based system that runs hierarchically from the domesticated crop, species, series/section, subgenus, genus to tribe and was proposed by Maxted et al. (2006). It operates on the principle that there exists a great relationship between taxonomic classification and relatedness and hence crossability (Maxted et al., 2006). Taxonomic classification in many plant families has however remained inconclusive, controversial and inconsistent (Huo et al., 2019; Yang et al., 2018) thus constraining the use of this approach. Unreliable and unstable taxonomic classification has potential to negatively impact global conservation efforts (Garnett & Christidis, 2017). Genomics is providing new capabilities characterized by greater power in taxonomic studies that is enabling taxonomic revision (Coates et al., 2018). Using genotyping by sequencing data, Wong et al. (2015) proposed a taxonomic revision in the *Lens* genus, proposing that *Lens* *odemensis* Ladiz should be considered as a distinct species and not a *subspecies* under *Lens* *culinaris*. There are, however, fears that the increased power and resolution of genome scans could lead to unnecessary splitting of species (Coates et al., 2018; Isaac et al., 2004) thus complicating conservation plans. Prioritization has enabled the development of inventory lists of priority CWRs which have proved to be a valuable resource that is guiding in situ conservation efforts in various countries (Khoury et al., 2013; Vincent et al., 2013). Prioritization is also useful to conservation practitioners and geneticists as it helps in identifying a reduced number of taxa which can be logistically feasible and affordable to be subjected to detailed genomic analysis.

## 5 SELECTION OF PRIORITY CONSERVATION SITES

In situ conservation is primarily undertaken through the establishment of in situ genetic reserves. Prior to undertaking any conservation actions, conservation managers need to undertake a systematic planning that involves both spatial and genetic prioritization of sites and CWR taxa. Different approaches have been used to identify the most suitable sites for ensuring efficient conservation of CWR diversity (Moray et al., 2014; Vincent et al., 2019). Ecogeographic and genetic diversity assessment surveys of CWRs provide knowledge on genetic diversity status and patterns which is vital in guiding conservation planning and ecosystem management (Navarro et al., 2017). Next generation sequencing is providing data that is helping in prioritization of in situ conservation sites in order to ensure cost effective conservation of high amounts of biodiversity (Flanagan et al., 2018). Population genomic analysis has capacity to enable the identification of biodiversity hotspots including hot spots of rare alleles. In addition to helping in identifying appropriate locations for in situ conservation, genomics is providing insights that are informing other conservation decisions such as sampling of genetic diversity for both in situ and ex situ conservation. For example, genome analysis has reported more genetic diversity in hotter and drier areas than in cooler and wetter regions (Fitzgerald et al., 2011; Shapter et al., 2013). These genetic diversity patterns are shaped by the prevailing environmental conditions as well as the existing biotic and abiotic stresses (Tso & Allan, 2019). This suggests that ecogeographic patterns can be used as a proxy for genetic diversity in the absence of genetic diversity data (Taylor et al., 2017). When assembling genetic resources for in situ and ex situ conservation, conservation practitioners may therefore want to put relatively more sampling efforts in the drier areas compared to the wet ones. Genomic analysis of taxonomic, phylogenetic and functional diversity patterns can guide the establishment of novel protected areas.

## 6 SPECIES IDENTIFICATION

A critical component of an in situ conservation strategy is undertaking of surveys and preparation of an inventory of CWRs in a particular country or geographical region. These surveys are underpinned by species identification and help provide information on species richness. Most conservation regulatory regimes, directives and plans recognize “species” as the primary unit of conservation (Coates et al., 2018). Accurate species identification however remains a major problem in many plant families and particularly among wild species. Cases of misidentification of CWRs in conservation have been reported (Baute et al., 2016; Mason et al., 2015; Ndjiondjop et al., 2018; Orjuela et al., 2014). Molecular taxonomic tools are enabling faster, cost effective and more accurate undertaking of surveys and inventories than traditional morphology based approaches (Thompson & Newmaster, 2014). Species discriminating markers are providing an efficient and cost effective method of plant identification. Species diagnostic SNP markers have been developed for discriminating *Oryza sativa* and *O*. *glaberrima* Steud from their CWRs, *O*. *barthii* A. Chevalier and *O*. *longistaminata* A. Chev. & Roehr (Ndjiondjop et al., 2018) as well as various CWRs in the *Citrus* genus (Curk et al., 2015). It has however been suggested that there is need to exercise caution when using GBS in identification of genotypes as it seems to have limited discriminatory capacity (Wong et al., 2015). The use of exome capture has been suggested to be faster and more reliable in species identification (Ogutcen et al., 2018). Genomic data complemented by geographical information has been used to identify cases of species misidentification in *Helianthus petiolaris* Nutt *and Helianthus bolanderi* A. Gray which are sunflower wild relatives (Baute et al., 2016).

DNA barcoding has emerged as a vital tool in supporting conservation. Several gene regions have previously been recommended to act as barcodes to assist in plant identification but their species discriminating capacity varies, with some performing suboptimally (CBOL Plant Working Group, 2009; Hollingsworth et al., 2016; Lahaye et al., 2008). The capacity of plastid genomes in plant identification has been demonstrated thus opening opportunities for it to potentially act as universal barcode (Nock et al., 2011). However, although the number of assembled chloroplast genomes is increasing rapidly (Daniell et al., 2016), it will require significant efforts by the scientific community to build a comprehensive chloroplast genome reference database. Once such a database is in place, chloroplast genome data is likely to become a tool that will allow authoritative species identification.

Advances in sequencing are now allowing portable, real time field based identification of closely related species using long read low coverage data obtained through nanopore sequencing technology (Parker et al., 2017). It might be debatable whether whole genome sequencing entirely for purposes of species identification is worth the efforts and cost as this might appear as data overkill. However, the data generated through this technology can be also be used for other applications such as phylogenomics (Parker et al., 2017). Other next generation sequencing based approaches such as genome skimming that can be used to generate nuclear genome‐based plant bar codes have been proposed (Hollingsworth et al., 2016). Due to the current advances in molecular taxonomy, it is now possible for researchers without specialist knowledge in plant taxonomy to accurately undertake species identification. Despite advances in DNA based species identification, morphological based approaches will continue playing a critical role in identification of species that are new to science.

## 7 MANAGING AND MONITORING IN SITU CWR GENETIC DIVERSITY

Active monitoring and management of in situ populations is critical in ensuring a successful conservation programme. However, most conservation sites do not have management plans targeting the CWRs. Routine assessment of genetic diversity of in situ populations is needed as it provides insights on amount and patterns of genetic variation in time and space at taxon and ecogeographical levels. This analysis provides a critical tool which guides conservation managers in making necessary management decisions. Advances in genomics are providing unprecedented opportunities of assessing genetic diversity at greater resolution than ever before.

### 7.1 Genomic application in monitoring indicators of genetic diversity

Loss of genetic diversity has for a long time mostly been described in abstract terms, as tools to monitor and quantify it have largely been lacking. Although there has been efforts to develop indicators of genetic erosion, not much progress has been made (FAO, 2010). It is important that conservation managers and other practitioners adopt techniques and strategies that help in monitoring trends in genetic diversity (Leroy et al., 2018) and to‐date, various such indicators have been developed or explored (Bruford et al., 2017; Fussi et al., 2016; Hoban et al., 2014; Khoury et al., 2019). With the current genomic revolution, there exists great potential for developing direct, comprehensive and robust indicators to monitor spatial and temporal patterns of genetic diversity at genome scale. However, despite its great potential, the use of molecular tools to monitor genetic diversity has largely been neglected. Proxies used to report genetic diversity changes such as number of accessions conserved or number of in situ populations are not a good indicator of genetic variation.

The concept of Essential Biodiversity Variable (EBS) was developed with the aim of providing a globally coordinated and harmonized system of biodiversity monitoring (Pereira et al., 2013). At the genetic level, allelic diversity has been proposed as one of the candidates for Essential Biodiversity Variable (EBS) (Pereira et al., 2013; Schmeller et al., 2018). Some of the potential direct genomic indicators of allelic diversity include number of alleles per loci and allele frequency. Genomic analysis with dense SNP marker data was found to have enough resolution to detect even subtle cases of genetic erosion (Hoban et al., 2014). Number of alleles has been found to have higher sensitivity in detecting genetic erosion than other genetic parameters (Hoban et al., 2014). Molecular analysis of allelic diversity in wild wheat and barley progenitor populations sampled after a period of 28 years revealed significant genetic divergence, with majority of the populations showing significant loss in allelic diversity while the others recorded allelic gains (Nevo et al., 2012). Significant improvement in genomic capacity including sequencing, genotyping and data analysis will probably be necessary if genomic tools are to be effectively deployed to monitor genetic diversity changes. Until the use of genomics as a tool to monitor genetic diversity changes is well developed, it might be necessary to prioritize the genomic loci to monitor. To make it practical, a monitoring system that uses genomic tools could, for example, be designed to give priority to monitoring changes in functional plant diversity loci. Priority could also be based on polymorphism levels, with priority being given to the less polymorphic loci within the population compared to the highly polymorphic ones. Scores of allelic diversity representativeness in both in situ and ex situ conservation could be developed.

Analysis of patterns and magnitude of various genetic related parameters such as population size, population structure, species richness and genetic diversity can help assess the impact of conservation actions and inform on necessary subsequent management interventions (FAO, 2017). Periodic genomic monitoring will help detect cases of genetic erosion and implement measures to arrest it thus contributing to the attainment of biodiversity conservation targets such as the Aichi Biodiversity Targets 2020 particularly Target 13. Target 13 states that “By 2020, the genetic diversity of cultivated plants and farmed and domesticated animals and of wild relatives, including other socioeconomically as well as culturally valuable species, is maintained, and strategies have been developed and implemented for minimizing genetic erosion and safeguarding their genetic diversity” (CBD, 2010). The issue of baseline data against which future diversity changes will be assessed is critically important if molecular tools are to be used for genetic diversity monitoring. Population genomic data of ex situ conserved accessions collected from a certain geographical region can be used as the baseline to monitor patterns of in situ genetic diversity over time in that particular region. Comparative genetic analysis of samples conserved ex situ with those found in situ (on‐farm) has revealed temporal changes in frequency of functionally important alleles (Vigouroux et al., 2011). This approach is likely to be less effective since the ex situ collection may not comprehensively represent all the available diversity for the taxa or population under study. It is likely for alleles not captured in the ex situ collection getting lost without the conservation manager ever noticing it. Clearly, genomic tools have the potential for developing a robust framework for monitoring and reporting genome‐based indicators.

### 7.2 Monitoring population structure

Conservation managers may need to monitor population structure in order to detect any changes as these could be caused by factors which might be of conservation concern. Population differentiation could be caused by gene flow, genetic drift or local adaptation (Shah et al., 2020). In addition to helping detect cases of population differentiation, genomics is enabling geneticists untangle the causes of the observed changes thus informing conservation management. Genomics has significantly advanced analysis of population structure to the extent of enabling detection of cryptic lineages (Steane et al., 2015). Detection of a cryptic taxa is of conservation importance particularly when its affects the performance of other species within a threatened habitat that needs to be conserved (Barbour et al., 2009). This is especially important if the different lineages require different management practices (Bickford et al., 2007). DArT sequencing of *Aegilops biuncialis* Vis, a wild relative of wheat, conducted by Ivanizs et al. (2019) and Sela et al. (2018) partitioned the accessions into five groups that were consistent with their ecogeographical origins. This analysis revealed four phenotypic categories of heading time that were largely associated with genetic structure and ecogeographic distribution. Analysis of population structure in cowpea (*Vigna unguiculata* (L) Walp landraces and wild relatives has provided insights on the crops domestication suggesting a divergent domestication process (Huynh et al., 2013). Similar insights were provided by the analysis of population structure in *O*. *barthii*, the progenitor of African rice (Wang et al., 2014). Genomic analysis has been used to study population structure of CWRs of various other crops among them rye (Schreiber et al., 2019), sunflower (Baute et al., 2016), lentil (Wong et al., 2015) and rice (Ndjiondjop et al., 2019). Population structure is important in revealing the extent of admixture as a result of gene flow between taxa and populations.

### 7.3 Identifying introgression and hybridization

Gene flow between crops and their wild relatives is of great conservation concern as it can have profound genetic impact on in situ populations. Consequences of gene flow include threat to genetic identity and integrity particularly of rare and endangered taxa, genetic erosion, weed invasiveness and escape of transgenes (Lu et al., 2016; Mondon et al., 2018). Although hybrids were previously considered undesirable and of little importance, there is increased recognition of their conservation value as they are a source of new genetic variation. It has been suggested that purposeful introgression and hybridization is a useful conservation management strategy particularly for those taxa and populations that are at risk of extinction due to poor adaptive capacity (Hamilton & Miller, 2016). Genome data has been used to study the extent and implications of gene flow (Mondon et al., 2018), particularly its role in shaping neutral and adaptive variation. It has been used to analyse introgression patterns between maize landraces, improved varieties and *Zea mays* ssp. *Mexicana*, a maize wild relative occurring in Mexico, revealing altered genetic diversity in maize landraces (Rojas‐Barrera et al., 2019). Genome data has been used to explain the emergence of eggplant accessions possessing “wild like” traits that represent hybrid swarm as a result of gene flow (Page et al., 2019). It has also been used to study gene flow in other crops among them wheat (Bernhardt et al., 2020; He et al., 2019) and sunflower (Mondon et al., 2018). Whole genome data is helping study introgression patterns thus providing useful insights which are important in untangling complex evolutionary and demographic history of species (Brozynska et al., 2017; Stein et al., 2018). Novel variation attributed to gene flow can enhance evolutionary potential particularly in changing climates.

By providing information on temporal and spatial patterns of admixture, genome data can help monitor the spread of any desirable and undesirable alleles. This may, for example, be useful for those alleles that may have exhibited signs of leading to the emergence of potentially invasive species. As genome editing becomes popular as a genomic tool for crop improvement, there is need to consider its genetic implications during conservation as edited genes are likely to move into CWRs through gene flow (Mondon et al., 2018). Biodiversity conservation will benefit from speciation genomics which helps analyse population divergence that leads to novel forms of biodiversity as a result of gene flow. CWRs which are isolated from potential sources of genetic contamination are particularly valuable for undertaking various genetic and genomic studies such as evolution as their genetic make‐up has not been altered by gene flow (Brozynska et al., 2017). The increased capacity of genomics to detect even subtle cases of genetic admixture has revealed the magnitude of the problem thereby raising concerns on its management (Wayne & Shaffer, 2016).

### 7.4 Plant reintroduction and enrichment planting

**FIGURE 3:**
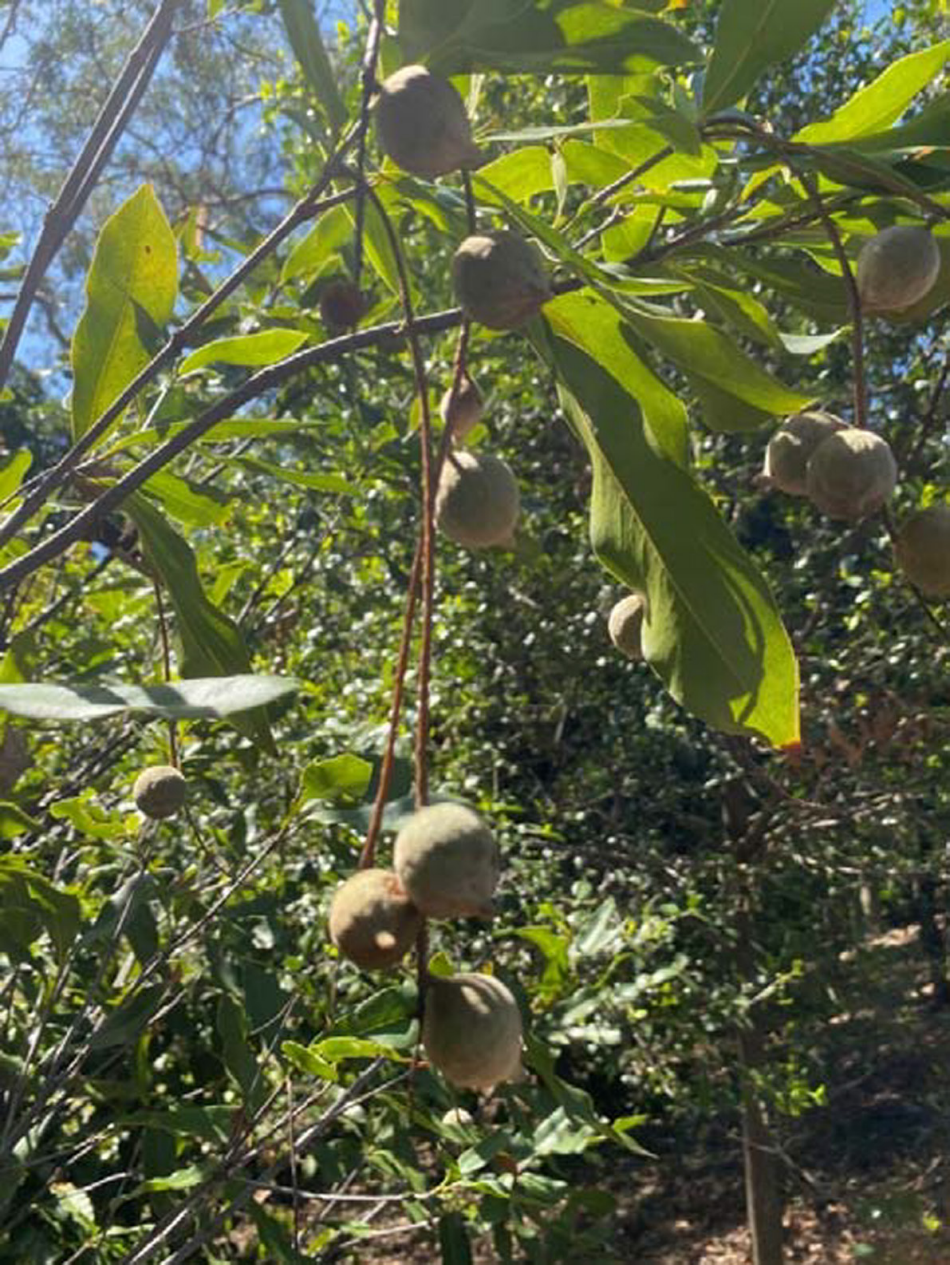
The population of *Macadamia jansenii*, an endangered wild relative of domesticated macadamia, has been characterized by genome sequencing (Sharma et al., 2021)

Due to various natural and anthropogenic causes, in situ populations may become threatened to the extent that their capacity to produce seed and regenerate is severely affected. Genomic analysis is providing useful information for supporting the conservation of these rare, threatened and endangered species (Figure 3). In some cases, such populations may need to be supported by introducing additional diversity to enrich the habitats. Plant reintroduction is also a common practice that is particularly useful in supporting the conservation of rare, threatened and vulnerable taxa or populations. The success of this management practice is however negatively affected by inadequate knowledge of the genetic diversity resident in the source population (Dalrymple et al., 2011; Godefroid et al., 2011). In both cases, the diversity can be sourced from ex situ conservation or naturally occurring populations. Where such materials are to be sourced from ex situ seed banks, genomics offers tools that help seed managers in selecting genetic material potentially possessing important traits that enhance their adaptive capacity (Wambugu et al., 2018). Genome wide analysis of functional diversity of the source population will help guide reintroduction efforts as it provides insights on the capacity of the reintroduced population to adapt (Breed et al., 2019; He et al., 2016). Knowledge on phenotype‐genotype relationships is increasing, with genetic markers associated with various traits being identified. Single nucleotide polymorphisms showing strong association with ecological adaptation have been reported (Parida et al., 2012) and provide a valuable resource that can potentially be used to select suitable materials. Enrichment planting material should possess high genetic diversity as this leads to greater adaptability and resilience of agroecosystems. Conservation managers can gauge the performance and fitness of the reintroduced populations by monitoring the allele frequencies of genetic variants (Shafer et al., 2015). Transcriptome sequencing will provide information on expressed genes and is therefore likely to be useful in adaptation and ecological genomics.

## 8 IDENTIFICATION OF ADAPTIVE LOCI

Adaptive loci refers to functional variation that determines the fitness of a taxa or population in a certain environment. Unlike genetic studies which have mainly employed presumably neutral markers, genomic studies have largely shifted to the use of genetic markers associated with functionally important diversity. Identification of this variation has been touted as the most important contribution that genomics can make in the conservation of biodiversity (McMahon et al., 2014). It is therefore not surprising that based on available literature, this appears to be the most studied area of conservation genomics. Numerous studies have reported on the capacity of genomic tools to identify adaptive traits and the genomic regions controlling them (Brunazzi et al., 2018; Exposito‐Alonso et al., 2018; Shah et al., 2020). Genomic analysis has enabled identification of genetic markers associated with certain environments in both cultivated and wild populations (Brunazzi et al., 2018). Cortés and Blair (2018) identified 115 SNPs and potential candidate genes associated with various bioclimatic variables related to drought in wild common bean accessions. Similarly, analysis of allelic diversity in drought related candidate genes in tepary bean and its wild relatives within the *Phaseolus acutifolius* A. Gray genepool, revealed six SNPs showing some association with environmental drought related variables (Buitrago‐Bitar et al., 2021). Knowledge on genome‐environment associations is vital in selecting genetic resources that can potentially adapt in target environments. Molecular analysis has revealed adaptive changes associated with emergence of novel alleles and increased allelic diversity in CWR populations over a period of time (Nevo et al., 2012; Vigouroux et al., 2011). These novel alleles play an important role in imparting local adaptation and should be prioritized for conservation both in situ and ex situ.

Common garden experiments employing a suite of genomic tools are a powerful approach for conducting local adaptation studies (de Villemereuil et al., 2016). Genome analysis of populations obtained from contrasting environments will reveal footprints of natural selection and hence help identify genomic loci that confers adaptive capacity against climate change. However, identifying adaptive loci from genome wide data may in some cases be problematic as signatures of adaptation may be difficult to detect (Vitti et al., 2013). Using SNP diversity indices such as nucleotide diversity (π) and Tajima’s D, it is possible to distinguish genomic signatures due to selection from those due to demographic processes as those due to adaptation are localized in certain regions while the others have genome wide distribution (Wakeley, 2008). There exists several methods and models for identifying loci involved in local adaptation by analysing differences in allele frequencies between populations obtained from geographical regions with contrasting environments. Some of these include BayEnv (Gautier, 2015; Günther & Coop, 2013), BayPass (Gautier, 2015) and BEDASSLE (Bradburd et al., 2013). These methods may help to identify geographical areas with unique variants or high levels of genetic diversity, both of which are important considerations when establishing in situ conservation sites.

Adaptive variation has mainly been identified at the nuclear genome but chloroplast genome analysis has recently revealed functional chloroplast genes that confer adaptive variation to diverse ecological conditions (Gao et al., 2019). Where information on adaptive variation is lacking for a particular taxa or population, genome scale SNP variation data may be used as a rough indicator of functional diversity and hence potential adaptability (He et al., 2016). It is predicted that due to climate changes, most species will shift their species range thus negatively impacting the suitability of current protected areas to conserve such species (Aguirre‐Gutiérrez et al., 2017; Beaumont et al., 2011; Pecl et al., 2017). Incorporating genomic data into models that predict the response of species to future climates has been found to increase the accuracy of the predictions (Razgour et al., 2019). Evolutionary genomics is providing great insights on plants response to climate change (Aguirre‐Liguori et al., 2021). Genomic analysis of genetic resources collected from regions possessing “future” climates might help identify loci necessary for imparting local adaptation in those regions. Transcriptome analysis is advancing knowledge and providing useful insights on the candidate genes and pathways likely to be associated with important adaptive traits in CWRs (Xu et al., 2014; Zeng et al., 2014). Such analysis has identified SNP markers associated with economically important traits. These trait‐linked or trait‐associated genetic markers are not only useful for crop improvement but also for guiding conservation actions. Once adaptive loci has been identified in CWRs, genomic tools can help introduce these adaptive alleles into domesticated crops in a targeted manner in order to increase adaptability.

## 9 ACCESS AND UTILIZATION OF IN SITU GENETIC DIVERSITY

In order to ensure success and sustainability of in situ CWR conservation, utilization of the conserved genetic resources should form an important component of the overall conservation strategy. One of the major challenges that have hindered the effective use of CWRs in research and crop improvement programmes is the inadequate understanding of their potential genetic value and unavailability of other important information. Genomics has expanded our understanding of the genetic architecture of functionally important traits. Some of these include leaf rust resistance in wheat and its wild relatives (Fatima et al., 2020; Liu et al., 2017), seed composition traits and adaptation to abiotic stresses in wild soybean (*Glycine soja*) (Anderson et al., 2016; Leamy et al., 2017) and local adaptation in teonsite, wild relative of maize (Pyhäjärvi et al., 2013). This includes the identification of genetic markers associated with such traits thus aiding in marker‐assisted selection. Domestication of new species presents another opportunity for enhancing the utilization of in situ CWR populations. While genomics has found great application in supporting the use of in situ genetic resources, this section is not intended to give a detailed review of this subject as it is beyond the scope of this paper. There are several reviews on the use of CWRs in crop improvement with the goal of enhancing food and nutritional security (Brozynska et al., 2015).

### 9.1 Accelerating domestication of new crops

Domestication marks a major deliberate effort of promoting the use of wild plants by manipulating their genetic and phenotypic traits in order to suit the needs of human kind. Genomics presents tools, resources and knowledge to accelerate domestication of new species thus potentially enhancing food and nutritional security (Brozynska et al., 2015). Genomics has enabled identification of various domestication genes and provided knowledge on functional importance of their allelic variation (Wambugu et al., 2021). These genomic advances are providing insights on genetic histories of important crops and the geographical spread of traits that may have been selected during domestication (Tripodi et al., 2021). Manipulation of domestication loci using current genomic technologies has potential of accelerating domestication of new species from CWR (Gasparini et al., 2021; Henry, 2012; Valle‐Echevarria et al., 2021). Genomics is for example assisting in the domestication and improvement of intermediate wheat grass (*Thinopyrum intermedium* L.) which is a perennial CWR of wheat (Kantarski et al., 2017; Zhang et al., 2016). Genome editing targeting six loci controlling agronomically important traits enabled the accelerated de novo domestication of wild tomato (*Solanum pimpinellifolium* L.) (Zsögön et al., 2018). In situ genetic reserves of CWRs increase availability of these taxa and presents an opportunity for human beings to interact, study and assess their food, nutritional, forage, medicinal and aesthetic value, which ultimately may lead to domestication. Deploying genomic resources such as trait‐associated SNPs to select genotypes possessing traits that are favoured by communities, breeders and researchers is likely to promote utilization and therefore enhance sustainability of the conservation programmes. The enhanced capacity in genome capacity is playing a major role in supporting plant biosecurity, particularly with regard to phytosanitary management.

### 9.2 Preventing misappropriation of in situ genetic diversity

Provisions that allow utilization of in situ CWR genetic diversity should be tempered with appropriate laws and regulations that prohibit illegal harvesting and over‐exploitation that could threaten the survival of the CWRs. Such regulatory frameworks should have sufficient and enforceable safeguards that helps prevent bio‐piracy. Molecular analysis is aiding law enforcement and regulatory authorities in detecting cases of illegal harvesting and trade of wild populations (Aubriot et al., 2013; Hartvig et al., 2015). Bio‐piracy remains a major ethical challenge that hinders effective conservation and efficient utilization of biodiversity. A key element of bio‐piracy is that access to genetic resources is not followed by fair and equitable sharing of benefits. Numerous cases of bio‐piracy have been reported around the world (Landon, 2007; Robinson, 2010). These cases have negatively impacted germplasm exchange by triggering the emergence of national laws in which countries are increasingly protecting and restricting access to their genetic resources. Next generation sequencing is providing tools that help in policing genetic resources against unauthorized access and misappropriation. Through genomics it is now possible to accurately identify the source of germplasm or other plant material.

It has been argued by Wambugu et al. (2018) that advances in genomics have potential for reversing this trend. Assurance that genomics has capacity to detect and successfully prosecute cases of illegal access to PGR might instil confidence in the germplasm exchange system thus potentially convincing countries to relax PGR access laws. On the flipside, there are real fears that advances in genomics and other scientific fields such as synthetic biology could lead to and fuel other forms of bio‐piracy or unequitable sharing of benefits. With capacity to synthesize DNA and for genome editing, it is possible to profit from the use of digital sequence information (DSI) even without accessing the material genetic resources. This would essentially mean that all existing access and benefit sharing (ABS) laws have been bypassed, and hence owners of the samples from which the DSI was obtained would not receive any benefits. Access to DSI remains largely unregulated. Despite the clear benefits of maintaining open access to DSI to research, trade regulation and food security (Cowell et al., 2022), some providers have strongly opposed such an unregulated access framework. Discussions are ongoing at the international policy level on the development of an acceptable framework on the governance of ABS for both genetic material and genomic data (Halewood et al., 2018; Sherman & Henry, 2021). There is need for an effective and transparent monitoring system that prevents digital bio piracy and ensures equitable benefits are paid to genetic resource providers (Sherman & Henry, 2021). An effective and efficient system might motivate genetic resource providers to put in place supportive mechanisms that allow unhindered access thus enhancing access, transfer and utilization of conserved germplasm. The fact that sequence data is sometimes not unique to a particular biological sample might make tracking complicated thereby making it difficult to determine who is rightfully entitled to receive the access benefits (Scholz et al., 2020). In relation to in situ conservation, it is important to put in place a system that ensures that funds obtained from access to genetic resources and associated sequence data are utilized to support conservation activities.

### 9.3 Breeding for climate resilience

Climate change currently poses the single largest threat to world agriculture and global food security. Although CWR populations are facing immense threats in the wild due to climate change, those populations that will successfully withstand these threats will be a useful source of genes for breeding for increased climate resilience. Genomics is providing tools to dissect the genetic basis of important traits and understand the functional trait diversity that is available in CWRS. Information on candidate genes and trait associated genetic markers is important in accelerating the use of CWRs in breeding programmes. The use of CWRs in crop improvement is particularly important in crops where genetic gain has been low as a result of inadequate variation in the domesticated gene pool for key target traits. Genomic data has enabled identification of regions that have lost important genetic diversity during the domestication process (Wang et al., 2009). This will facilitate targeted reintroduction of this diversity into cultivated taxa. The greatest contribution of CWRs in crop improvement is in imparting biotic and abiotic resistance (Dempewolf et al., 2017). Some of the significant contributions of CWRs in crop breeding include the introduction of resistance to late blight from wild potato *Solanum demissum* Lindl and stem rust from wild wheat *Aegilops tauschii* Coss into the respective cultivated gene pool (Prescott‐Allen & Prescott‐Allen, 1986). In situ conservation is important in supporting breeding using CWRs by not only increasing availability of these resources but also imparting the necessary adaptive traits through the evolutionary processes that take place in different ecogeographical areas.

## 10 CHALLENGES AND FUTURE PROSPECTS

One of the major challenges facing in situ conservation is that the majority of CWRs are poorly studied and may not have a lot of genomic information and resources which may be required to guide conservation management. For species with limited genomic information, there have been considerable advances in genomic technologies allowing genome wide marker discovery, even for poorly studied taxa. Genotyping by sequencing is a powerful marker technology which is suitable for species with no reference genome sequence (Elshire et al., 2011). Inadequate knowledge on various aspects of relevance to conservation has continued to hinder conservation efforts. For example, information on available functional variation and its genetic underpinnings is critical in selecting adapted germplasm but is lacking for many CWRs. Information on genome environment associations (GEA) is not available for a vast majority of CWRs, making it difficult to match CWR populations with the right environment for in situ conservation.

The cost, computational requirements and necessary bioinformatic expertise for deploying genomics is high and may be beyond the reach of most conservation practitioners thus hindering its real world application (Flanagan et al., 2018). Although sequencing costs have reduced dramatically over the last decade, genome‐wide analysis, particularly of a large number of samples, is still not affordable for resource constrained conservation initiatives. As we continue to transition from genetic studies based on few molecular markers to genome‐wide analysis, selection of correct study designs, data analysis and interpretations thereof are emerging as significant challenges (Wambugu et al., 2019). Although there appears to be general agreement that genomics presents a promising tool to support conservation, there is a debate as to whether the field of conservation genomics has matured enough to find real application into conservation practice (Shafer et al., 2015). Many policy makers and conservation managers have failed to recognize and appreciate the potential of genomics in conservation and management of biodiversity (Hunter et al., 2018). Moreover, it has been acknowledged that genomics might in some cases not lead to improved conservation management practices (Allendorf et al., 2010), Funding and inadequate genomic expertise among conservation practitioners also remain major hindrances to the widespread application of genomics in conservation. Training programmes on the use of genomics in conservation should be developed for conservation managers. The field of genomics is evolving rapidly and there is no telling what the future holds in terms of its capacity and application in conservation.

In future, genome editing might offer an opportunity to support PGR conservation by introducing novel alleles that confer adaptive capacity against climate change as well as removing deleterious ones that reduce fitness (Breed et al., 2019; Johnsson et al., 2019; Supple & Shapiro, 2018). However, despite its potential, genome editing carries with its risks and dangers that should be carefully considered before deploying it in PGR conservation. This might particularly be helpful for those species that are seriously threatened by environmental stresses or general lack of adaptation capacity. Public policies on biodiversity conservation and agricultural production are often contradictory, with differing stakeholders and government agencies being responsible. Genomics is widely used in crop improvement and is increasingly used in biodiversity management. Genomics of CWRs may have suffered from these competing perspectives in policy and funding for genomics tools, with CWRs being seen as a biodiversity problem for agriculture policy makers and an agricultural issue by biodiversity policy makers. If these interest groups are able to collaborate, it creates the opportunity for policy designed to support biodiversity and policies aiming to support agriculture and food security to be harmonized around use of genomic analysis to support conservation and utilization. An increased alignment of policy would ensure progress in the conservation of CWRs to support food security.

## CONFLICTS OF INTERESTS

The authors declare no conflict of interest.

## AUTHOR CONTRIBUTIONS

Peterson W. Wambugu wrote the first draft. Both authors contributed to the editing of the manuscript.

## Data Availability

Data archiving is not applicable to this review.

## References

[mec16402-bib-0001] Aguirre‐Gutiérrez, J. , van Treuren, R. , Hoekstra, R. , & van Hintum, T. J. L. (2017). Crop wild relatives range shifts and conservation in Europe under climate change. Diversity and Distributions, 23(7), 739–750. 10.1111/ddi.12573

[mec16402-bib-0002] Aguirre‐Liguori, J. A. , Ramírez‐Barahona, S. , & Gaut, B. S. (2021). The evolutionary genomics of species’ responses to climate change. Nature Ecology & Evolution, 5(10), 1350–1360. 10.1038/s41559-021-01526-9 34373621

[mec16402-bib-0003] Allendorf, F. W. , Hohenlohe, P. A. , & Luikart, G. (2010). Genomics and the future of conservation genetics. Nature Reviews: Genetics, 11(10), 697–709. 10.1038/nrg2844 20847747

[mec16402-bib-0004] Anderson, J. E. , Kono, T. J. , Stupar, R. M. , Kantar, M. B. , & Morrell, P. L. (2016). Environmental association analyses identify candidates for abiotic stress tolerance in glycine soja, the wild progenitor of cultivated soybeans. G3 (Bethesda), 6(4), 835–843. 10.1534/g3.116.026914 26818076 PMC4825654

[mec16402-bib-0005] Aubriot, X. , Lowry, P. P. 2nd , Cruaud, C. , Couloux, A. , & Haevermans, T. (2013). DNA barcoding in a biodiversity hot spot: Potential value for the identification of Malagasy Euphorbia L. listed in CITES Appendices I and II. Molecular Ecology Resources, 13(1), 57–65. 10.1111/1755-0998.12028 23095939

[mec16402-bib-0006] Barbour, R. C. , O’Reilly‐Wapstra, J. M. , Little, D. W. D. , Jordan, G. J. , Steane, D. A. , Humphreys, J. R. , Bailey, J. K. , Whitham, T. G. , & Potts, B. M. (2009). A geographic mosaic of genetic variation within a foundation tree species and its community‐level consequences. Ecology, 90(7), 1762–1772. 10.1890/08-0951.1 19694126

[mec16402-bib-0007] Bari, A. , Street, K. , Mackay, M. , Endresen, D. T. F. , De Pauw, E. , & Amri, A. (2012). Focused identification of germplasm strategy (FIGS) detects wheat stem rust resistance linked to environmental variables. Genetic Resources and Crop Evolution, 59(7), 1465–1481. 10.1007/s10722-011-9775-5

[mec16402-bib-0008] Baute, G. J. (2015). Genomics of sunflower improvement: From wild relatives to a global oil seed. The University of British Columbia.

[mec16402-bib-0009] Baute, G. J. , Owens, G. L. , Bock, D. G. , & Rieseberg, L. H. (2016). Genome‐wide genotyping‐by‐sequencing data provide a high‐resolution view of wild Helianthus diversity, genetic structure, and interspecies gene flow. American Journal of Botany, 103(12), 2170–2177. 10.3732/ajb.1600295 27965242

[mec16402-bib-0010] Beaumont, L. J. , Pitman, A. , Perkins, S. , Zimmermann, N. E. , Yoccoz, N. G. , & Thuiller, W. (2011). Impacts of climate change on the world’s most exceptional ecoregions. Proceedings of the National Academy of Sciences, 108(6), 2306–2311. 10.1073/pnas.1007217108 PMC3038729 21262825

[mec16402-bib-0011] Bernhardt, N. , Brassac, J. , Dong, X. , Willing, E.‐M. , Poskar, C. H. , Kilian, B. , & Blattner, F. R. (2020). Genome‐wide sequence information reveals recurrent hybridization among diploid wheat wild relatives. The Plant Journal, 102(3), 493–506. 10.1111/tpj.14641 31821649

[mec16402-bib-0012] Bhullar, N. K. , Street, K. , Mackay, M. , Yahiaoui, N. , Keller, B. , & Bennetzen, J. L. (2009). Unlocking wheat genetic resources for the molecular identification of previously undescribed functional alleles at the “Pm3” resistance locus. Proceedings of the National Academy of Sciences of the United States of America, 106(23), 9519–9524. 10.1073/pnas.0904152106 19470492 PMC2686639

[mec16402-bib-0013] Bickford, D. , Lohman, D. J. , Sodhi, N. S. , Ng, P. K. L. , Meier, R. , Winker, K. , Ingram, K. K. , & Das, I. (2007). Cryptic species as a window on diversity and conservation. Trends in Ecology & Evolution, 22(3), 148–155. 10.1016/j.tree.2006.11.004 17129636

[mec16402-bib-0014] Bilz, M. , Kell, S. P. , Maxted, N. , & Lansdown, R. V. (2011). European red list of vascular plants. European Commission.

[mec16402-bib-0015] Bradburd, G. S. , Ralph, P. L. , & Coop, G. M. (2013). Disentangling the effects of geographic and ecological isolation on genetic differentiation. Evolution, 67(11), 3258–3273. 10.1111/evo.12193 24102455 PMC3808528

[mec16402-bib-0016] Breed, M. F. , Harrison, P. A. , Blyth, C. , Byrne, M. , Gaget, V. , Gellie, N. J. C. , Groom, S. V. C. , Hodgson, R. , Mills, J. G. , Prowse, T. A. A. , Steane, D. A. , & Mohr, J. J. (2019). The potential of genomics for restoring ecosystems and biodiversity. Nature Reviews Genetics, 20(10), 615–628. 10.1038/s41576-019-0152-0 31300751

[mec16402-bib-0017] Brozynska, M. , Copetti, D. , Furtado, A. , Wing, R. A. , Crayn, D. , Fox, G. , Ishikawa, R. , & Henry, R. J. (2017). Sequencing of Australian wild rice genomes reveals ancestral relationships with domesticated rice. Plant Biotechnology Journal, 15(6), 765–774. 10.1111/pbi.12674 27889940 PMC5425390

[mec16402-bib-0018] Brozynska, M. , Furtado, A. , & Henry, R. J. (2015). Genomics of crop wild relatives: Expanding the gene pool for crop improvement. Plant Biotechnology Journal, 14, 1070–1085. 10.1111/pbi.12454 26311018 PMC11389173

[mec16402-bib-0019] Brozynska, M. , Omar, E. S. , Furtado, A. , Crayn, D. , Simon, B. , Ishikawa, R. , & Henry, R. J. (2014). Chloroplast genome of novel rice germplasm identified in Northern Australia. Tropical Plant Biology, 7(3), 111–120. 10.1007/s12042-014-9142-8 25485030 PMC4245483

[mec16402-bib-0020] Bruford, M. W. , Davies, N. , Dulloo, M. E. , Faith, D. P. , & Walters, M. (2017). Monitoring changes in genetic diversity. In M. Walters , & R. J. Scholes (Eds.), The GEO handbook on biodiversity observation networks (pp. 107–128). Springer International Publishing. 10.1007/978-3-319-27288-7_5

[mec16402-bib-0021] Brunazzi, A. , Scaglione, D. , Talini, R. F. , Miculan, M. , Magni, F. , Poland, J. , & Dell’Acqua, M. (2018). Molecular diversity and landscape genomics of the crop wild relative *Triticum urartu* across the fertile crescent. The Plant Journal, 94(4), 670–684. 10.1111/tpj.13888 29573496

[mec16402-bib-0022] Buitrago‐Bitar, M. A. , Cortés, A. J. , López‐Hernández, F. , Londoño‐Caicedo, J. M. , Muñoz‐Florez, J. E. , Muñoz, L. C. , & Blair, M. W. (2021). Allelic diversity at abiotic stress responsive genes in relationship to ecological drought indices for cultivated tepary bean, *Phaseolus acutifolius* A. Gray, and its wild relatives. Genes (Basel), 12(4), 556. https://www.mdpi.com/2073‐4425/12/4/556 33921270 10.3390/genes12040556 PMC8070098

[mec16402-bib-0023] Cadotte, M. W. , & Tucker, C. M. (2018). Difficult decisions: Strategies for conservation prioritization when taxonomic, phylogenetic and functional diversity are not spatially congruent. Biological Conservation, 225, 128–133. 10.1016/j.biocon.2018.06.014

[mec16402-bib-0024] Carbonell‐Caballero, J. , Alonso, R. , Ibañez, V. , Terol, J. , Talon, M. , & Dopazo, J. (2015). A phylogenetic analysis of 34 chloroplast genomes elucidates the relationships between wild and domestic species within the genus citrus. Molecular Biology and Evolution, 32(8), 2015–2035. 10.1093/molbev/msv082 25873589 PMC4833069

[mec16402-bib-0025] Castañeda‐Álvarez, N. P. , Khoury, C. K. , Achicanoy, H. A. , Bernau, V. , Dempewolf, H. , Eastwood, R. J. , Guarino, L. , Harker, R. H. , Jarvis, A. , Maxted, N. , Müller, J. V. , Ramirez‐Villegas, J. , Sosa, C. C. , Struik, P. C. , Vincent, H. , & Toll, J. (2016). Global conservation priorities for crop wild relatives. Nature Plants, 2(4), 16022. 10.1038/nplants.2016.22 27249561

[mec16402-bib-0026] CBD . (2010). Stategic plan for biodiversity 2011‒2020 and the Aichi targets. https://www.cbd.int/doc/strategic‐plan/2011‐2020/Aichi‐Targets‐EN.pdf

[mec16402-bib-0027] CBOL Plant Working Group . (2009). A DNA barcode for land plants. Proceedings of the National Academy of Sciences of the United States of America, 106(31), 12794–12797. 10.1073/pnas.0905845106 19666622 PMC2722355

[mec16402-bib-0028] Coates, D. J. , Byrne, M. , & Moritz, C. (2018). Genetic diversity and conservation units: Dealing with the species‐population continuum in the age of genomics [Review]. Frontiers in Ecology and Evolution, 6(165). 10.3389/fevo.2018.00165

[mec16402-bib-0029] Cortés, A. J. , & Blair, M. W. (2018). Genotyping by sequencing and genome‐environment associations in wild common bean predict widespread divergent adaptation to drought. Frontiers in Plant Science, 9, 128. 10.3389/fpls.2018.00128 29515597 PMC5826387

[mec16402-bib-0030] Cowell, C. , Paton, A. , Borrell, J. S. , Williams, C. , Wilkin, P. , Antonelli, A. , Baker, W. J. , Buggs, R. , Fay, M. F. , Gargiulo, R. , Grace, O. M. , Kuhnhäuser, B. G. , Woudstra, Y. , & Kersey, P. J. (2022). Uses and benefits of digital sequence information from plant genetic resources: Lessons learnt from botanical collections. Plants, People, Planet, 4(1), 33–43. 10.1002/ppp3.10216

[mec16402-bib-0031] Curk, F. , Ancillo, G. , Ollitrault, F. , Perrier, X. , Jacquemoud‐Collet, J.‐P. , Garcia‐Lor, A. , Navarro, L. , & Ollitrault, P. (2015). Nuclear species‐diagnostic SNP markers mined from 454 amplicon sequencing reveal admixture genomic structure of modern citrus varieties. PLoS One, 10(5), e0125628. 10.1371/journal.pone.0125628 25973611 PMC4431842

[mec16402-bib-0032] Dalrymple, S. , Stewart, G. , & Pullin, A. (2011). Are reintroductions an effective way of mitigating against plant extinctions? CEE review 07–008 (SR32). Collaboration for Environmental Evidence. www.environmentalevidence.org/SR32.html

[mec16402-bib-0033] Daniell, H. , Lin, C. S. , Yu, M. , & Chang, W. J. (2016). Chloroplast genomes: Diversity, evolution, and applications in genetic engineering. Genome Biology, 17(1), 134. 10.1186/s13059-016-1004-2 27339192 PMC4918201

[mec16402-bib-0034] de Villemereuil, P. , Gaggiotti, O. E. , Mouterde, M. , & Till‐Bottraud, I. (2016). Common garden experiments in the genomic era: New perspectives and opportunities. Heredity, 116(3), 249–254. 10.1038/hdy.2015.93 26486610 PMC4806574

[mec16402-bib-0035] Dempewolf, H. , Baute, G. , Anderson, J. , Kilian, B. , Smith, C. , & Guarino, L. (2017). Past and future use of wild relatives in crop breeding. Crop Science, 57(3), 1070–1082. 10.2135/cropsci2016.10.0885

[mec16402-bib-0036] Dempewolf, H. , Eastwood, R. J. , Guarino, L. , Khoury, C. K. , Müller, J. V. , & Toll, J. (2014). Adapting agriculture to climate change: A global initiative to collect, conserve, and use crop wild relatives. Agroecology and Sustainable Food Systems, 38(4), 369–377. 10.1080/21683565.2013.870629

[mec16402-bib-0501] Elshire, R. J. , Glaubitz, J. C. , Sun, Q. , Poland, J. A. , Kawamoto, J. A. , Buckler, E. S. , & Mitchell, S. E. (2011). A robust, simple genotyping‐by‐sequencing (GBS) approach for high diversity species. PLOS One. 10.1371/journal.pone.0019379 PMC3087801 21573248

[mec16402-bib-0037] Endresen, D. T. F. , Street, K. , Mackay, M. , Bari, A. , Amri, A. , De Pauw, E. , Nazari, K. , & Yahyaoui, A. (2012). Sources of resistance to stem rust (Ug99) in bread wheat and durum wheat identified using focused identification of germplasm strategy. Crop Science, 52(2), 764–773. 10.2135/cropsci2011.08.0427

[mec16402-bib-0038] Exposito‐Alonso, M. , Drost, H.‐G. , Burbano, H. A. , & Weigel, D. (2020). The Earth BioGenome project: opportunities and challenges for plant genomics and conservation. The Plant Journal, 102(2), 222–229. 10.1111/tpj.14631 31788877

[mec16402-bib-0039] Exposito‐Alonso, M. , Vasseur, F. , Ding, W. , Wang, G. , Burbano, H. A. , & Weigel, D. (2018). Genomic basis and evolutionary potential for extreme drought adaptation in Arabidopsis thaliana. Nature Ecology & Evolution, 2(2), 352–358. 10.1038/s41559-017-0423-0 29255303 PMC5777624

[mec16402-bib-0040] FAO . (2010). Second report on the world’s plant genetic resources for food and agriculture. FAO.

[mec16402-bib-0041] FAO . (2017). Voluntary guidelines for the conservation and sustainable use of crop wild relatives and wild food plants. Food and Agriculture Organization of the United Nations.

[mec16402-bib-0042] Fatima, F. , McCallum, B. D. , Pozniak, C. J. , Hiebert, C. W. , McCartney, C. A. , Fedak, G. , You, F. M. , & Cloutier, S. (2020). Identification of new leaf rust resistance loci in wheat and wild relatives by array‐based SNP genotyping and association genetics [Original Research]. Frontiers in Plant Science, 11. 10.3389/fpls.2020.583738 PMC7701059 33304363

[mec16402-bib-0043] Fielder, H. , Brotherton, P. , Hosking, J. , Hopkins, J. J. , Ford‐Lloyd, B. , & Maxted, N. (2015). Enhancing the conservation of crop wild relatives in England. PLoS One, 10(6), e0130804. 10.1371/journal.pone.0130804 26110773 PMC4481409

[mec16402-bib-0044] Fitzgerald, T. L. , Shapter, F. M. , McDonald, S. , Waters, D. L. E. , Chivers, I. H. , Drenth, A. , Nevo, E. , & Henry, R. J. (2011). Genome diversity in wild grasses under environmental stress. Proceedings of the National Academy of Sciences USA, 108(52), 21140–21145. 10.1073/pnas.1115203108 PMC3248542 22173638

[mec16402-bib-0045] Flanagan, S. P. , Forester, B. R. , Latch, E. K. , Aitken, S. N. , & Hoban, S. (2018). Guidelines for planning genomic assessment and monitoring of locally adaptive variation to inform species conservation. Evolutionary Applications, 11(7), 1035–1052. 10.1111/eva.12569 30026796 PMC6050180

[mec16402-bib-0046] Fussi, B. , Westergren, M. , Aravanopoulos, F. , Baier, R. , Kavaliauskas, D. , Finzgar, D. , Alizoti, P. , Bozic, G. , Avramidou, E. , Konnert, M. , & Kraigher, H. (2016). Forest genetic monitoring: An overview of concepts and definitions. Environmental Monitoring and Assessment, 188(8), 493. 10.1007/s10661-016-5489-7 27473107 PMC4967086

[mec16402-bib-0047] Gao, L.‐Z. , Liu, Y.‐L. , Zhang, D. , Li, W. , Gao, J. U. , Liu, Y. , Li, K. , Shi, C. , Zhao, Y. , Zhao, Y.‐J. , Jiao, J.‐Y. , Mao, S.‐Y. , Gao, C.‐W. , & Eichler, E. E. (2019). Evolution of Oryza chloroplast genomes promoted adaptation to diverse ecological habitats. Communications Biology, 2(1), 278. 10.1038/s42003-019-0531-2 31372517 PMC6659635

[mec16402-bib-0048] Garnett, S. , & Christidis, L. (2017). Taxonomy anarchy hampers conservation. Nature, 546, 25–27. 10.1038/546025a 28569833

[mec16402-bib-0049] Gasparini, K. , Moreira, J. D. R. , Peres, L. E. P. , & Zsögön, A. (2021). De novo domestication of wild species to create crops with increased resilience and nutritional value. Current Opinion in Plant Biology, 60. 10.1016/j.pbi.2021.102006 33556879

[mec16402-bib-0050] Gautier, M. (2015). Genome‐wide scan for adaptive divergence and association with population‐specific covariates. Genetics, 201(4), 1555–1579. 10.1534/genetics.115.181453 26482796 PMC4676524

[mec16402-bib-0051] Godefroid, S. , Piazza, C. , Rossi, G. , Buord, S. , Stevens, A.‐D. , Aguraiuja, R. , Cowell, C. , Weekley, C. W. , Vogg, G. , Iriondo, J. M. , Johnson, I. , Dixon, B. , Gordon, D. , Magnanon, S. , Valentin, B. , Bjureke, K. , Koopman, R. , Vicens, M. , Virevaire, M. , & Vanderborght, T. (2011). How successful are plant species reintroductions? Biological Conservation, 144(2), 672–682. 10.1016/j.biocon.2010.10.003

[mec16402-bib-0052] Günther, T. , & Coop, G. (2013). Robust identification of local adaptation from allele frequencies. Genetics, 195(1), 205–220. 10.1534/genetics.113.152462 23821598 PMC3761302

[mec16402-bib-0053] Hajjar, R. , & Hodgkin, T. (2007). The use of wild relatives in crop improvement: A survey of developments over the last 20 years. Euphytica, 156(1–2), 1–13. 10.1007/s10681-007-9363-0

[mec16402-bib-0054] Halewood, M. , Lopez Noriega, I. , Ellis, D. , Roa, C. , Rouard, M. , & Sackville Hamilton, R. (2018). Using genomic sequence information to increase conservation and sustainable use of crop diversity and benefit‐sharing. Biopreservation and Biobanking, 16(5), 368–376. 10.1089/bio.2018.0043 30325667 PMC6204560

[mec16402-bib-0055] Hamilton, J. A. , & Miller, J. M. (2016). Adaptive introgression as a resource for management and genetic conservation in a changing climate. Conservation Biology, 30(1), 33–41. 10.1111/cobi.12574 26096581

[mec16402-bib-0056] Harlan, J. R. , & de Wet, J. M. J. (1971). Toward a rational classification of cultivated plants. Taxon, 20(4), 509–517. 10.2307/1218252

[mec16402-bib-0057] Hartvig, I. , Czako, M. , Kjær, E. D. , Nielsen, L. R. , & Theilade, I. (2015). The use of DNA barcoding in identification and conservation of rosewood (*Dalbergia* spp.). PLoS One, 10(9), e0138231. 10.1371/journal.pone.0138231 26375850 PMC4573973

[mec16402-bib-0058] He, F. , Pasam, R. , Shi, F. , Kant, S. , Keeble‐Gagnere, G. , Kay, P. , Forrest, K. , Fritz, A. , Hucl, P. , Wiebe, K. , Knox, R. , Cuthbert, R. , Pozniak, C. , Akhunova, A. , Morrell, P. L. , Davies, J. P. , Webb, S. R. , Spangenberg, G. , Hayes, B. , … Akhunov, E. (2019). Exome sequencing highlights the role of wild‐relative introgression in shaping the adaptive landscape of the wheat genome. Nature Genetics, 51(5), 896–904. 10.1038/s41588-019-0382-2 31043759

[mec16402-bib-0059] He, X. , Johansson, M. L. , & Heath, D. D. (2016). Role of genomics and transcriptomics in selection of reintroduction source populations. Conservation Biology, 30(5), 1010–1018. 10.1111/cobi.12674 26756292

[mec16402-bib-0060] Henry, R. J. (2012). Next‐generation sequencing for understanding and accelerating crop domestication. Briefings in Functional Genomics, 11(1), 51–56. 10.1093/bfgp/elr032 22025450

[mec16402-bib-0061] Henry, R. J. (2013). Sequencing of wild crop relatives to support the conservation and utilization of plant genetic resources. Plant Genetic Resources, 12(S1), S9–S11. 10.1017/S1479262113000439

[mec16402-bib-0062] Hoban, S. , Arntzen, J. A. , Bruford, M. W. , Godoy, J. A. , Rus Hoelzel, A. , Segelbacher, G. , Vilà, C. , & Bertorelle, G. (2014). Comparative evaluation of potential indicators and temporal sampling protocols for monitoring genetic erosion. Evolutionary Applications, 7(9), 984–998. 10.1111/eva.12197 25553062 PMC4231590

[mec16402-bib-0063] Hollingsworth, P. M. , Li, D. Z. , van der Bank, M. , & Twyford, A. D. (2016). Telling plant species apart with DNA: From barcodes to genomes. Philosophical Transactions of the Royal Society of London. Series B, Biological Sciences, 371(1702), 20150338. 10.1098/rstb.2015.0338 27481790 PMC4971190

[mec16402-bib-0064] Hunter, M. E. , Hoban, S. M. , Bruford, M. W. , Segelbacher, G. , & Bernatchez, L. (2018). Next‐generation conservation genetics and biodiversity monitoring. Evolutionary Applications, 11(7), 1029–1034. 10.1111/eva.12661 30026795 PMC6050179

[mec16402-bib-0065] Huo, Y. M. , Gao, L. M. , Liu, B. J. , Yang, Y. Y. , Kong, S. P. , Sun, Y. Q. , Yang, Y. H. , & Wu, X. (2019). Complete chloroplast genome sequences of four Allium species: comparative and phylogenetic analyses. Scientific Reports, 9(1), 12250. 10.1038/s41598-019-48708-x 31439882 PMC6706373

[mec16402-bib-0066] Huynh, B.‐L. , Close, T. J. , Roberts, P. A. , Hu, Z. , Wanamaker, S. , Lucas, M. R. , Chiulele, R. , Cissé, N. , David, A. , Hearne, S. , Fatokun, C. , Diop, N. N. , & Ehlers, J. D. (2013). Gene pools and the genetic architecture of domesticated cowpea. The Plant Genome, 6(3). 10.3835/plantgenome2013.03.0005

[mec16402-bib-0067] Hwang, E.‐Y. , Wei, H. , Schroeder, S. G. , Fickus, E. W. , Quigley, C. V. , Elia, P. , & Song, Q. (2019). Genetic diversity and phylogenetic relationships of annual and perennial *Glycine* species. G3: Genes|Genomes|Genetics, 9(7), 2325–2336. 10.1534/g3.119.400220 31097479 PMC6643897

[mec16402-bib-0068] Isaac, N. J. B. , Mallet, J. , & Mace, G. M. (2004). Taxonomic inflation: Its influence on macroecology and conservation. Trends in Ecology & Evolution, 19(9), 464–469. 10.1016/j.tree.2004.06.004 16701308

[mec16402-bib-0069] Ivanizs, L. , Monostori, I. , Farkas, A. , Megyeri, M. , Mikó, P. , Türkösi, E. , Gaál, E. , Lenykó‐Thegze, A. , Szőke‐Pázsi, K. , Szakács, É. , Darkó, É. , Kiss, T. , Kilian, A. , & Molnár, I. (2019). Unlocking the genetic diversity and population structure of a wild gene source of wheat, *Aegilops biuncialis* Vis., and its relationship with the heading time [Original Research]. Frontiers in Plant Science, 10(1531). 10.3389/fpls.2019.01531 PMC6882925 31824545

[mec16402-bib-0070] Jarvis, A. , Lane, A. , & Hijmans, R. J. (2008). The effect of climate change on crop wild relatives. Agriculture, Ecosystems & Environment, 126(1), 13–23. 10.1016/j.agee.2008.01.013

[mec16402-bib-0071] Johnsson, M. , Gaynor, R. C. , Jenko, J. , Gorjanc, G. , de Koning, D.‐J. , & Hickey, J. M. (2019). Removal of alleles by genome editing (RAGE) against deleterious load. Genetics Selection Evolution, 51(1), 14. 10.1186/s12711-019-0456-8 PMC6472060 30995904

[mec16402-bib-0072] Kantarski, T. , Larson, S. , Zhang, X. , DeHaan, L. , Borevitz, J. , Anderson, J. , & Poland, J. (2017). Development of the first consensus genetic map of intermediate wheatgrass (*Thinopyrum intermedium*) using genotyping‐by‐sequencing. Theoretical and Applied Genetics, 130(1), 137–150. 10.1007/s00122-016-2799-7 27738715

[mec16402-bib-0073] Khoury, C. K. , Amariles, D. , Soto, J. S. , Diaz, M. V. , Sotelo, S. , Sosa, C. C. , Ramírez‐Villegas, J. , Achicanoy, H. A. , Velásquez‐Tibatá, J. , Guarino, L. , León, B. , Navarro‐Racines, C. , Castañeda‐Álvarez, N. P. , Dempewolf, H. , Wiersema, J. H. , & Jarvis, A. (2019). Comprehensiveness of conservation of useful wild plants: An operational indicator for biodiversity and sustainable development targets. Ecological Indicators, 98, 420–429. 10.1016/j.ecolind.2018.11.016

[mec16402-bib-0074] Khoury, C. K. , Greene, S. , Wiersema, J. , Maxted, N. , Jarvis, A. , & Struik, P. C. (2013). An inventory of crop wild relatives of the United States. Crop Science, 53(4), 1496–1508. 10.2135/cropsci2012.10.0585

[mec16402-bib-0075] Koo, B. , Pardey, P. G. , & Wright, B. D. (2003). The economic costs of conserving genetic resources at the CGIAR centers⋆. Agricultural Economics, 29(3), 287–297. 10.1111/j.1574-0862.2003.tb00165.x

[mec16402-bib-0076] Labokas, J. , Maxted, N. , Kell, S. , Brehm, J. M. , & Iriondo, J. M. (2018). Development of national crop wild relative conservation strategies in European countries. Genetic Resources and Crop Evolution, 65(5), 1385–1403. 10.1007/s10722-018-0621-x

[mec16402-bib-0077] Lahaye, R. , van der Bank, M. , Bogarin, D. , Warner, J. , Pupulin, F. , Gigot, G. , Maurin, O. , Duthoit, S. , Barraclough, T. G. , & Savolainen, V. (2008). DNA barcoding the floras of biodiversity hotspots. Proceedings of the National Academy of Sciences of the United States of America, 105(8), 2923–2928. 10.1073/pnas.0709936105 18258745 PMC2268561

[mec16402-bib-0078] Laikre, L. (2010). Genetic diversity is overlooked in international conservation policy implementation. Conservation Genetics, 11(2), 349–354. 10.1007/s10592-009-0037-4

[mec16402-bib-0079] Landon, A. J. (2007). Bioprospecting and biopiracy in Latin America: The case of Maca in Perú. Nebraska Anthropologist, 32.

[mec16402-bib-0080] Leamy, L. J. , Zhang, H. , Li, C. , Chen, C. Y. , & Song, B.‐H. (2017). A genome‐wide association study of seed composition traits in wild soybean (Glycine soja). BMC Genomics, 18(1), 18. 10.1186/s12864-016-3397-4 28056769 PMC5217241

[mec16402-bib-0081] Leroy, G. , Carroll, E. L. , Bruford, M. W. , DeWoody, J. A. , Strand, A. , Waits, L. , & Wang, J. (2018). Next‐generation metrics for monitoring genetic erosion within populations of conservation concern. Evolutionary Applications, 11(7), 1066–1083. 10.1111/eva.12564 30026798 PMC6050182

[mec16402-bib-0082] Liu, W. , Maccaferri, M. , Chen, X. , Laghetti, G. , Pignone, D. , Pumphrey, M. , & Tuberosa, R. (2017). Genome‐wide association mapping reveals a rich genetic architecture of stripe rust resistance loci in emmer wheat (*Triticum turgidum* ssp. dicoccum). Theoretical and Applied Genetics, 130(11), 2249–2270. 10.1007/s00122-017-2957-6 28770301 PMC5641275

[mec16402-bib-0083] Lu, B.‐R. , Yang, X. , & Ellstrand, N. C. (2016). Fitness correlates of crop transgene flow into weedy populations: A case study of weedy rice in China and other examples. Evolutionary Applications, 9(7), 857–870. 10.1111/eva.12377 27468304 PMC4947148

[mec16402-bib-0084] Mason, A. S. , Zhang, J. , Tollenaere, R. , Vasquez Teuber, P. , Dalton‐Morgan, J. , Hu, L. , Yan, G. , Edwards, D. , Redden, R. , & Batley, J. (2015). High‐throughput genotyping for species identification and diversity assessment in germplasm collections. Molecular Ecology Resources, 15(5), 1091–1101. 10.1111/1755-0998.12379 25641370

[mec16402-bib-0085] Maxted, N. , Dulloo, E. , V Ford‐Lloyd, B. , Iriondo, J. M. , & Jarvis, A. (2008). Gap analysis: A tool for complementary genetic conservation assessment. Diversity and Distributions, 14(6), 1018–1030. 10.1111/j.1472-4642.2008.00512.x

[mec16402-bib-0086] Maxted, N. , Ford‐Lloyd, B. V. , Jury, S. , Kell, S. , & Scholten, M. (2006). Towards a definition of a crop wild relative. Biodiversity & Conservation, 15(8), 2673–2685. 10.1007/s10531-005-5409-6

[mec16402-bib-0087] Maxted, N. , & Kell, S. (2009). Establishment of a global network for the in situ conservation of crop wild relatives: Status and needs. Commission on Genetic Resources for Food and Agriculture (CGRFA), 266.

[mec16402-bib-0088] McMahon, B. J. , Teeling, E. C. , & Höglund, J. (2014). How and why should we implement genomics into conservation? Evolutionary Applications, 7(9), 999–1007. 10.1111/eva.12193 25553063 PMC4231591

[mec16402-bib-0089] Meilleur, B. A. , & Hodgkin, T. (2004). In situ conservation of crop wild relatives: Status and trends. Biodiversity and Conservation, 13(4), 663–684. 10.1023/B:BIOC.0000011719.03230.17

[mec16402-bib-0090] Mondon, A. , Owens, G. L. , Poverene, M. , Cantamutto, M. , & Rieseberg, L. H. (2018). Gene flow in Argentinian sunflowers as revealed by genotyping‐by‐sequencing data. Evolutionary Applications, 11(2), 193–204. 10.1111/eva.12527 29387155 PMC5775495

[mec16402-bib-0091] Moray, C. , Game, E. T. , & Maxted, N. (2014). Prioritising in situ conservation of crop resources: A case study of African cowpea (*Vigna unguiculata*). Scientific Reports, 4(1), 5247. 10.1038/srep05247 24936740 PMC4060501

[mec16402-bib-0092] Navarro, L. M. , Fernández, N. , Guerra, C. , Guralnick, R. , Kissling, W. D. , Londoño, M. C. , Muller‐Karger, F. , Turak, E. , Balvanera, P. , Costello, M. J. , Delavaud, A. , El Serafy, G. Y. , Ferrier, S. , Geijzendorffer, I. , Geller, G. N. , Jetz, W. , Kim, E.‐S. , Kim, H. J. , Martin, C. S. , … Pereira, H. M. (2017). Monitoring biodiversity change through effective global coordination. Current Opinion in Environmental Sustainability, 29, 158–169. 10.1016/j.cosust.2018.02.005

[mec16402-bib-0093] Ndjiondjop, M. N. , Alachiotis, N. , Pavlidis, P. , Goungoulou, A. , Kpeki, S. B. , Zhao, D. , & Semagn, K. (2019). Comparisons of molecular diversity indices, selective sweeps and population structure of African rice with its wild progenitor and Asian rice. Theoretical and Applied Genetics, 132(4), 1145–1158. 10.1007/s00122-018-3268-2 30578434 PMC6449321

[mec16402-bib-0094] Ndjiondjop, M. N. , Semagn, K. , Zhang, J. , Gouda, A. C. , Kpeki, S. B. , Goungoulou, A. , Wambugu, P. , Dramé, K. N. , Bimpong, I. K. , & Zhao, D. (2018). Development of species diagnostic SNP markers for quality control genotyping in four rice (*Oryza L*.) species. Molecular Breeding: New Strategies in Plant Improvement, 38(11), 131. 10.1007/s11032-018-0885-z 30416368 PMC6208651

[mec16402-bib-0095] Nevo, E. , Fu, Y.‐B. , Pavlicek, T. , Khalifa, S. , Tavasi, M. , & Beiles, A. (2012). Evolution of wild cereals during 28 years of global warming in Israel. Proceedings of the National Academy of Sciences, 109(9), 3412–3415. 10.1073/pnas.1121411109 PMC3295258 22334646

[mec16402-bib-0096] Nock, C. J. , Waters, D. L. E. , Edwards, M. A. , Bowen, S. G. , Rice, N. , Cordeiro, G. M. , & Henry, R. J. (2011). Chloroplast genome sequences from total DNA for plant identification. Plant Biotechnology Journal, 9(3), 328–333. 10.1111/j.1467-7652.2010.00558.x 20796245

[mec16402-bib-0097] Ogutcen, E. , Ramsay, L. , von Wettberg, E. B. , & Bett, K. E. (2018). Capturing variation in lens (Fabaceae): Development and utility of an exome capture array for lentil. Applications in Plant Sciences, 6(7), e01165. 10.1002/aps3.1165 30131907 PMC6055568

[mec16402-bib-0098] Orjuela, J. , Sabot, F. , Chéron, S. , Vigouroux, Y. , Adam, H. , Chrestin, H. , Sanni, K. , Lorieux, M. , & Ghesquière, A. (2014). An extensive analysis of the African rice genetic diversity through a global genotyping. Theoretical and Applied Genetics, 127(10), 2211–2223. 10.1007/s00122-014-2374-z 25119871

[mec16402-bib-0099] Page, A. , Gibson, J. , Meyer, R. S. , & Chapman, M. A. (2019). Eggplant domestication: Pervasive gene flow, feralization, and transcriptomic divergence. Molecular Biology and Evolution, 36(7), 1359–1372. 10.1093/molbev/msz062 31039581

[mec16402-bib-0100] Pardey, P. G. , Koo, B. , Wright, B. D. , Van Dusen, M. E. , Skovmand, B. , & Taba, S. (2001). Costing the conservation of genetic resources: CIMMYT’s ex situ maize and wheat collection. Crop Science, 41(4), 1286–1299. 10.2135/cropsci2001.4141286x

[mec16402-bib-0101] Parida, S. K. , Mukerji, M. , Singh, A. K. , Singh, N. K. , & Mohapatra, T. (2012). SNPs in stress‐responsive rice genes: Validation, genotyping, functional relevance and population structure. BMC Genomics, 13(1), 426. 10.1186/1471-2164-13-426 22921105 PMC3562522

[mec16402-bib-0102] Parker, J. , Helmstetter, A. J. , Devey, D. , Wilkinson, T. , & Papadopulos, A. S. T. (2017). Field‐based species identification of closely‐related plants using real‐time nanopore sequencing. Scientific Reports, 7(1), 8345. 10.1038/s41598-017-08461-5 28827531 PMC5566789

[mec16402-bib-0103] Pecl, G. T. , Araújo, M. B. , Bell, J. D. , Blanchard, J. , Bonebrake, T. C. , Chen, I.‐C. , Clark, T. D. , Colwell, R. K. , Danielsen, F. , Evengård, B. , Falconi, L. , Ferrier, S. , Frusher, S. , Garcia, R. A. , Griffis, R. B. , Hobday, A. J. , Janion‐Scheepers, C. , Jarzyna, M. A. , Jennings, S. , … Williams, S. E. (2017). Biodiversity redistribution under climate change: Impacts on ecosystems and human well‐being. Science, 355(6332), eaai9214. 10.1126/science.aai9214 28360268

[mec16402-bib-0104] Pereira, H. M. , Ferrier, S. , Walters, M. , Geller, G. N. , Jongman, R. H. G. , Scholes, R. J. , Bruford, M. W. , Brummitt, N. , Butchart, S. H. M. , Cardoso, A. C. , Coops, N. C. , Dulloo, E. , Faith, D. P. , Freyhof, J. , Gregory, R. D. , Heip, C. , Höft, R. , Hurtt, G. , Jetz, W. , … Wegmann, M. (2013). Essential biodiversity variables. Science, 339(6117), 277–278. 10.1126/science.1229931 23329036

[mec16402-bib-0105] Phillips, J. (2017). Development of crop wild relative conservation strategies for Norway. The University of Birmingham.

[mec16402-bib-0106] Prescott‐Allen, C. , & Prescott‐Allen, R. (1986). First resource wild species in the North American economy. Yale University Press. 10.2307/j.ctt211qvck

[mec16402-bib-0107] Pyhäjärvi, T. , Hufford, M. B. , Mezmouk, S. , & Ross‐Ibarra, J. (2013). Complex patterns of local adaptation in teosinte. Genome Biology and Evolution, 5(9), 1594–1609. 10.1093/gbe/evt109 23902747 PMC3787665

[mec16402-bib-0108] Razgour, O. , Forester, B. , Taggart, J. B. , Bekaert, M. , Juste, J. , Ibáñez, C. , Puechmaille, S. J. , Novella‐Fernandez, R. , Alberdi, A. , & Manel, S. (2019). Considering adaptive genetic variation in climate change vulnerability assessment reduces species range loss projections. Proceedings of the National Academy of Sciences, 116(21), 10418–10423. 10.1073/pnas.1820663116 PMC6535011 31061126

[mec16402-bib-0109] Robinson, D. F. (2010). Confronting biopiracy: Challenges, cases and international debates (Vol. 1). Routledge.

[mec16402-bib-0110] Rojas‐Barrera, I. C. , Wegier, A. , Sánchez González, J. D. J. , Owens, G. L. , Rieseberg, L. H. , & Piñero, D. (2019). Contemporary evolution of maize landraces and their wild relatives influenced by gene flow with modern maize varieties. Proceedings of the National Academy of Sciences, 116(42), 21302–21311. 10.1073/pnas.1817664116 PMC6800366 31570572

[mec16402-bib-0111] Schmeller, D. S. , Weatherdon, L. V. , Loyau, A. , Bondeau, A. , Brotons, L. , Brummitt, N. , Geijzendorffer, I. R. , Haase, P. , Kuemmerlen, M. , Martin, C. S. , Mihoub, J.‐B. , Rocchini, D. , Saarenmaa, H. , Stoll, S. , & Regan, E. C. (2018). A suite of essential biodiversity variables for detecting critical biodiversity change. Biological Reviews, 93(1), 55–71. 10.1111/brv.12332 28447398

[mec16402-bib-0112] Scholz, A. H. , Hillebrand, U. , Freitag, J. , Devanshi, S. , Seitz, C. , Thiele, T. , & Van Zimmeren, E. (2020). Finding compromise on ABS & DSI in the CBD: Requirements & policy ideas from a scientific perspective. 10.13140/RG.2.2.35180.80001

[mec16402-bib-0113] Schreiber, M. , Himmelbach, A. , Börner, A. , & Mascher, M. (2019). Genetic diversity and relationship between domesticated rye and its wild relatives as revealed through genotyping‐by‐sequencing. Evolutionary Applications, 12(1), 66–77. 10.1111/eva.12624 30622636 PMC6304746

[mec16402-bib-0114] Sela, H. , Ezrati, S. , & Olivera, P. D. (2018). Genetic diversity of three Israeli wild relatives of wheat from the Sitopsis section of Aegilops. Israel Journal of Plant Sciences, 65(3–4), 161–174. 10.1163/22238980-00001059

[mec16402-bib-0115] Shafer, A. B. A. , Wolf, J. B. W. , Alves, P. C. , Bergström, L. , Bruford, M. W. , Brännström, I. , Colling, G. , Dalén, L. , De Meester, L. , Ekblom, R. , Fawcett, K. D. , Fior, S. , Hajibabaei, M. , Hill, J. A. , Hoezel, A. R. , Höglund, J. , Jensen, E. L. , Krause, J. , Kristensen, T. N. , … Zieliński, P. (2015). Genomics and the challenging translation into conservation practice. Trends in Ecology & Evolution, 30(2), 78–87. 10.1016/j.tree.2014.11.009 25534246

[mec16402-bib-0116] Shah, N. , Wakabayashi, T. , Kawamura, Y. , Skovbjerg, C. K. , Wang, M.‐Z. , Mustamin, Y. , Isomura, Y. , Gupta, V. , Jin, H. , Mun, T. , Sandal, N. , Azuma, F. , Fukai, E. , Seren, Ü. , Kusakabe, S. , Kikuchi, Y. , Nitanda, S. , Kumaki, T. , Hashiguchi, M. , … Andersen, S. U. (2020). Extreme genetic signatures of local adaptation during Lotus japonicus colonization of Japan. Nature Communications, 11(1), 253. 10.1038/s41467-019-14213-y PMC6959357 31937774

[mec16402-bib-0117] Shapter, F. M. , Cross, M. , Ablett, G. , Malory, S. , Chivers, I. H. , King, G. J. , & Henry, R. J. (2013). High‐throughput sequencing and mutagenesis to accelerate the domestication of Microlaena stipoides as a new food crop. PLoS One, 8(12), e82641. 10.1371/journal.pone.0082641 24367532 PMC3867367

[mec16402-bib-0118] Sharma, P. , Murigneux, V. , Haimovitz, J. , Nock, C. J. , Tian, W. , Kharabian Masouleh, A. , & Henry, R. J. (2021). The genome of the endangered Macadamia jansenii displays little diversity but represents an important genetic resource for plant breeding. Plant Direct, 5(12), e364. 10.1002/pld3.364 34938939 PMC8671617

[mec16402-bib-0119] Sherman, B. , & Henry, R. J. (2021). Access to biodiversity for food production: Reconciling open access digital sequence information with access and benefit sharing. Molecular Plant, 14(5), 701–704. 10.1016/j.molp.2021.03.005 33684540

[mec16402-bib-0120] Small, R. L. , Cronn, R. C. , & Wendel, J. F. (2004). Use of nuclear genes for phylogeny reconstruction in plants. Australian Systematic Botany, 17(2), 145–170. 10.1071/SB03015

[mec16402-bib-0121] Song, Y. , Chen, Y. , Lv, J.‐Z. , Xu, J. , Zhu, S. , & Li, M. (2019). Comparative chloroplast genomes of sorghum species: Sequence divergence and phylogenetic relationships. BioMed Research International, 2019. 10.1155/2019/5046958 PMC6444266 31016191

[mec16402-bib-0122] Steane, D. A. , Potts, B. M. , McLean, E. , Collins, L. , Prober, S. M. , Stock, W. D. , Vaillancourt, R. E. , & Byrne, M. (2015). Genome‐wide scans reveal cryptic population structure in a dry‐adapted eucalypt. Tree Genetics & Genomes, 11(3), 33. 10.1007/s11295-015-0864-z

[mec16402-bib-0123] Stein, J. C. , Yu, Y. , Copetti, D. , Zwickl, D. J. , Zhang, L. I. , Zhang, C. , Chougule, K. , Gao, D. , Iwata, A. , Goicoechea, J. L. , Wei, S. , Wang, J. , Liao, Y. I. , Wang, M. , Jacquemin, J. , Becker, C. , Kudrna, D. , Zhang, J. , Londono, C. E. M. , … Wing, R. A. (2018). Genomes of 13 domesticated and wild rice relatives highlight genetic conservation, turnover and innovation across the genus Oryza. Nature Genetics, 50(2), 285–296. 10.1038/s41588-018-0040-0 29358651

[mec16402-bib-0124] Supple, M. A. , & Shapiro, B. (2018). Conservation of biodiversity in the genomics era. Genome Biology, 19(1), 131. 10.1186/s13059-018-1520-3 30205843 PMC6131752

[mec16402-bib-0125] Syfert, M. M. , Castañeda‐Álvarez, N. P. , Khoury, C. K. , Särkinen, T. , Sosa, C. C. , Achicanoy, H. A. , & Knapp, S. (2016). Crop wild relatives of the brinjal eggplant (*Solanum melongena*): Poorly represented in genebanks and many species at risk of extinction. American Journal of Botany, 103(4), 635–651. 10.3732/ajb.1500539 27026215

[mec16402-bib-0126] Takahashi, H. , Sato, Y.‐I. , & Nakamura, I. (2008). Evolutionary analysis of two plastid DNA sequences in cultivated and wild species of Oryza. Breeding Science, 58, 225–233. 10.1270/jsbbs.58.225

[mec16402-bib-0127] Taylor, N. G. , Kell, S. P. , Holubec, V. , Parra‐Quijano, M. , Chobot, K. , & Maxted, N. (2017). A systematic conservation strategy for crop wild relatives in the Czech Republic. Diversity and Distributions, 23(4), 448–462. 10.1111/ddi.12539

[mec16402-bib-0128] Teso, M. L. , Lamas, E. , Parra‐Quijano, M. , de la Rosa, L. , Fajardo, J. , & Iriondo, J. M. (2018). National inventory and prioritization of crop wild relatives in Spain. Genetic Resources and Crop Evolution, 65(4), 1237–1253. 10.1007/s10722-018-0610-0

[mec16402-bib-0129] Thompson, K. A. , & Newmaster, S. G. (2014). Molecular taxonomic tools provide more accurate estimates of species richness at less cost than traditional morphology‐based taxonomic practices in a vegetation survey. Biodiversity and Conservation, 23(6), 1411–1424. 10.1007/s10531-014-0672-z

[mec16402-bib-0130] Tripodi, P. , Rabanus‐Wallace, M. T. , Barchi, L. , Kale, S. , Esposito, S. , Acquadro, A. , & Stein, N. (2021). Global range expansion history of pepper (*Capsicum* spp.) revealed by over 10,000 genebank accessions. Proceedings of the National Academy of Sciences, 118(34), e2104315118. 10.1073/pnas.2104315118 PMC8403938 34400501

[mec16402-bib-0131] Tso, K. L. , & Allan, G. J. (2019). Environmental variation shapes genetic variation in *Bouteloua gracilis*: Implications for restoration management of natural populations and cultivated varieties in the southwestern United States. Ecology and Evolution, 9(1), 482–499. 10.1002/ece3.4767 30680130 PMC6342110

[mec16402-bib-0132] Valle‐Echevarria, A. D. , Fumia, N. , Gore, M. A. , & Kantar, M. (2021). Accelerating crop domestication in the era of gene editing. In I. Goldman (Ed.), Plant breeding reviews (pp. 185–211). Wiley. 10.1002/9781119828235.ch4

[mec16402-bib-0133] Varshney, R. K. , Roorkiwal, M. , Sun, S. , Bajaj, P. , Chitikineni, A. , Thudi, M. , Singh, N. P. , Du, X. , Upadhyaya, H. D. , Khan, A. W. , Wang, Y. , Garg, V. , Fan, G. , Cowling, W. A. , Crossa, J. , Gentzbittel, L. , Voss‐Fels, K. P. , Valluri, V. K. , Sinha, P. , … Liu, X. (2021). A chickpea genetic variation map based on the sequencing of 3,366 genomes. Nature, 599(7886), 622–627. 10.1038/s41586-021-04066-1 34759320 PMC8612933

[mec16402-bib-0134] Vigouroux, Y. , Mariac, C. , De Mita, S. , Pham, J.‐L. , Gérard, B. , Kapran, I. , Sagnard, F. , Deu, M. , Chantereau, J. , Ali, A. , Ndjeunga, J. , Luong, V. , Thuillet, A.‐C. , Saïdou, A.‐A. , & Bezançon, G. (2011). Selection for earlier flowering crop associated with climatic variations in the Sahel. PLoS One, 6(5), e19563. 10.1371/journal.pone.0019563 21573243 PMC3087796

[mec16402-bib-0135] Vincent, H. , Amri, A. , Castañeda‐Álvarez, N. P. , Dempewolf, H. , Dulloo, E. , Guarino, L. , Hole, D. , Mba, C. , Toledo, A. , & Maxted, N. (2019). Modeling of crop wild relative species identifies areas globally for in situ conservation. Communications Biology, 2(1), 136. 10.1038/s42003-019-0372-z 31044161 PMC6478866

[mec16402-bib-0136] Vincent, H. , Wiersema, J. , Kell, S. , Fielder, H. , Dobbie, S. , Castañeda‐Álvarez, N. P. , Guarino, L. , Eastwood, R. , Leόn, B. , & Maxted, N. (2013). A prioritized crop wild relative inventory to help underpin global food security. Biological Conservation, 167, 265–275. 10.1016/j.biocon.2013.08.011

[mec16402-bib-0137] Vitti, J. J. , Grossman, S. R. , & Sabeti, P. C. (2013). Detecting natural selection in genomic data. Annual Review of Genetics, 47, 97–120. 10.1146/annurev-genet-111212-133526 24274750

[mec16402-bib-0138] Wakeley, J. (2008). Coalescent theory: An introduction. Harvard University.

[mec16402-bib-0139] Wambugu, P. W. , Brozynska, M. , Furtado, A. , Waters, D. L. , & Henry, R. J. (2015). Relationships of wild and domesticated rices (Oryza AA genome species) based upon whole chloroplast genome sequences. Scientific ReporTS, 5, 13957. 10.1038/srep13957 26355750 PMC4564799

[mec16402-bib-0140] Wambugu, P. , Furtado, A. , Waters, D. , Nyamongo, D. , & Henry, R. (2013). Conservation and utilization of African Oryza genetic resources. Rice, 6(1), 29. 10.1186/1939-8433-6-29 24280189 PMC4883696

[mec16402-bib-0141] Wambugu, P. W. , Ndjiondjop, M.‐N. , & Henry, R. J. (2018). Role of genomics in promoting the utilization of plant genetic resources in genebanks. Briefings in Functional Genomics, 17(3), 198–206. 10.1093/bfgp/ely014 29688255 PMC5967547

[mec16402-bib-0142] Wambugu, P. W. , Ndjiondjop, M.‐N. , & Henry, R. (2019). Advances in molecular genetics and genomics of African rice (*Oryza glaberrima Steud*). Plants (Basel, Switzerland), 8(10), 376. 10.3390/plants8100376 PMC6843444 31561516

[mec16402-bib-0143] Wambugu, P. W. , Ndjiondjop, M.‐N. , & Henry, R. (2021). Genetics and genomics of African rice (*Oryza glaberrima Steud*) domestication. Rice, 14(1), 6. 10.1186/s12284-020-00449-6 33415579 PMC7790969

[mec16402-bib-0144] Wang, L. , Hao, L. , Li, X. , Hu, S. , Ge, S. , & Yu, J. (2009). SNP deserts of Asian cultivated rice: Genomic regions under domestication. Journal of Evolutionary Biology, 22(4), 751–761. 10.1111/j.1420-9101.2009.01698.x 19243488

[mec16402-bib-0145] Wang, M. , Yu, Y. , Haberer, G. , Marri, P. R. , Fan, C. , Goicoechea, J. L. , Zuccolo, A. , Song, X. , Kudrna, D. , Ammiraju, J. S. S. , Cossu, R. M. , Maldonado, C. , Chen, J. , Lee, S. , Sisneros, N. , de Baynast, K. , Golser, W. , Wissotski, M. , Kim, W. , … Wing, R. A. (2014). The genome sequence of African rice (*Oryza glaberrima*) and evidence for independent domestication. Nature Genetics, 46(9), 982–988. 10.1038/ng.3044 25064006 PMC7036042

[mec16402-bib-0146] Wang, W. , Mauleon, R. , Hu, Z. , Chebotarov, D. , Tai, S. , Wu, Z. , Li, M. , Zheng, T. , Fuentes, R. R. , Zhang, F. , Mansueto, L. , Copetti, D. , Sanciangco, M. , Palis, K. C. , Xu, J. , Sun, C. , Fu, B. , Zhang, H. , Gao, Y. , … Leung, H. (2018). Genomic variation in 3,010 diverse accessions of Asian cultivated rice. Nature, 557(7703), 43–49. 10.1038/s41586-018-0063-9 29695866 PMC6784863

[mec16402-bib-0147] Waters, D. L. E. , Nock, C. J. , Ishikawa, R. , Rice, N. , & Henry, R. J. (2012). Chloroplast genome sequence confirms distinctness of Australian and Asian wild rice. Ecology and Evolution, 2(1), 211–217. 10.1002/ece3.66 22408737 PMC3297189

[mec16402-bib-0148] Wayne, R. K. , & Shaffer, H. B. (2016). Hybridization and endangered species protection in the molecular era. Molecular Ecology, 25(11), 2680–2689. 10.1111/mec.13642 27064931

[mec16402-bib-0149] Wong, M. M. L. , Gujaria‐Verma, N. , Ramsay, L. , Yuan, H. Y. , Caron, C. , Diapari, M. , Vandenberg, A. , & Bett, K. E. (2015). Classification and characterization of species within the genus lens using genotyping‐by‐sequencing (GBS). PLoS One, 10(3), e0122025. 10.1371/journal.pone.0122025 25815480 PMC4376907

[mec16402-bib-0150] Xu, W. , Li, R. , Zhang, N. , Ma, F. , Jiao, Y. , & Wang, Z. (2014). Transcriptome profiling of *Vitis amurensis*, an extremely cold‐tolerant Chinese wild Vitis species, reveals candidate genes and events that potentially connected to cold stress. Plant Molecular Biology, 86(4–5), 527–541. 10.1007/s11103-014-0245-2 25190283

[mec16402-bib-0151] Yang, Z. , Zhao, T. , Ma, Q. , Liang, L. , & Wang, G. (2018). Comparative genomics and phylogenetic analysis revealed the chloroplast genome variation and interspecific relationships of Corylus (Betulaceae) species. Frontiers in Plant Science, 9, 927. 10.3389/fpls.2018.00927 30038632 PMC6046460

[mec16402-bib-0152] Zeng, J. , He, X. , Wu, D. , Zhu, B. O. , Cai, S. , Nadira, U. A. , Jabeen, Z. , & Zhang, G. (2014). Comparative transcriptome profiling of two Tibetan wild barley genotypes in responses to low potassium. PLoS One, 9(6), e100567. 10.1371/journal.pone.0100567 24949953 PMC4065039

[mec16402-bib-0153] Zhang, X. , Sallam, A. , Gao, L. , Kantarski, T. , Poland, J. , DeHaan, L. R. , Wyse, D. L. , & Anderson, J. A. (2016). Establishment and optimization of genomic selection to accelerate the domestication and improvement of intermediate wheatgrass. Plant Genome, 9(1). 10.3835/plantgenome2015.07.0059 27898759

[mec16402-bib-0154] Zhang, Y. Y. , Liu, F. , Tian, N. , Che, J. R. , Sun, X. L. , Lai, Z. X. , & Cheng, C. Z. (2019). Characterization of the complete chloroplast genome of Sanming wild banana (*Musa itinerans*) and phylogenetic relationships. Mitochondrial DNA B Resource, 4(2), 2614–2616. 10.1080/23802359.2019.1642167 PMC7706688 33365650

[mec16402-bib-0155] Zsögön, A. , Čermák, T. , Naves, E. R. , Notini, M. M. , Edel, K. H. , Weinl, S. , Freschi, L. , Voytas, D. F. , Kudla, J. , & Peres, L. E. P. (2018). De novo domestication of wild tomato using genome editing. Nature Biotechnology, 36(12), 1211–1216. 10.1038/nbt.4272 30272678

